# Prevalence and risk factors analysis of postpartum depression at early stage using hybrid deep learning model

**DOI:** 10.1038/s41598-024-54927-8

**Published:** 2024-02-24

**Authors:** Umesh Kumar Lilhore, Surjeet Dalal, Neeraj Varshney, Yogesh Kumar Sharma, K. B. V. Brahma Rao, V. V. R. Maheswara Rao, Roobaea Alroobaea, Sarita Simaiya, Martin Margala, Prasun Chakrabarti

**Affiliations:** 1 Department of Computer Science & Engineering, Chandigarh University Gharuan Mohali, Gharuan, 140413 Punjab India; 2 Amity School of Engineering and Technology, Amity University Haryana, Panchgaon, Haryana India; 3 Department of Computer Engineering and Applications GLA University, Mathura, India; 4 Department of Computer Science & Engineering, Koneru Lakshmaiah Education Foundation, Greenfield, Vaddeswaram, Guntur, Andhra Pradesh India; 5 Computer Science and Engineering, Koneru Lakshmaiah Education Foundation, Vaddeswaram, Guntur, Andhra Pradesh India; 6 Dept. of Computer Science and Engineering, Shri Vishnu Engineering College for Women (A), Bhimavaram, Andhra Pradesh India 534202; 7 Department of Computer Science, College of Computers and Information Technology, Taif University, P. O. Box 11099, 21944 Taif, Saudi Arabia; 8 Department of Computer Science and Engineering, Chandigarh University, Mohali, Punjab, 140413 India; 9 School of Computing and Informatics, University of Louisiana, Lafayette, USA; 10 Department of Computer Science and Engineering, Sir Padampat Singhania University, Udaipur, 313601 Rajasthan India

**Keywords:** Postpartum Depression Disorder, Attention-Based method, Improved Bi-LSTM, CNN, LSTM, Deep learning, Machine learning, Transfer learning, Diseases, Health care, Medical research

## Abstract

Postpartum Depression Disorder (PPDD) is a prevalent mental health condition and results in severe depression and suicide attempts in the social community. Prompt actions are crucial in tackling PPDD, which requires a quick recognition and accurate analysis of the probability factors associated with this condition. This concern requires attention. The primary aim of our research is to investigate the feasibility of anticipating an individual's mental state by categorizing individuals with depression from those without depression using a dataset consisting of text along with audio recordings from patients diagnosed with PPDD. This research proposes a hybrid PPDD framework that combines Improved Bi-directional Long Short-Term Memory (IBi-LSTM) with Transfer Learning (TL) based on two Convolutional Neural Network (CNN) architectures, respectively CNN-text and CNN audio. In the proposed model, the CNN section efficiently utilizes TL to obtain crucial knowledge from text and audio characteristics, whereas the improved Bi-LSTM module combines written material and sound data to obtain intricate chronological interpersonal relationships. The proposed model incorporates an attention technique to augment the effectiveness of the Bi-LSTM scheme. An experimental analysis is conducted on the PPDD online textual and speech audio dataset collected from UCI. It includes textual features such as age, women's health tracks, medical histories, demographic information, daily life metrics, psychological evaluations, and ‘speech records’ of PPDD patients. Data pre-processing is applied to maintain the data integrity and achieve reliable model performance. The proposed model demonstrates a great performance in better precision, recall, accuracy, and F1-score over existing deep learning models, including VGG-16, Base-CNN, and CNN-LSTM. These metrics indicate the model's ability to differentiate among women at risk of PPDD vs. non-PPDD. In addition, the feature importance analysis demonstrates that specific risk factors substantially impact the prediction of PPDD. The findings of this research establish a basis for improved precision and promptness in assessing the risk of PPDD, which may ultimately result in earlier implementation of interventions and the establishment of support networks for women who are susceptible to PPDD.

## Introduction

Postpartum Depression Disorder (PPDD) is a mood disorder that manifests in women after childbirth, poses a significant obstacle to maternal mental health. This phenomenon negatively impacts a crucial and transformative stage in a woman's life, reducing the positive experiences associated with motherhood and affecting the individual in question and the family. Although PPDD is a mental health issue commonly observed, the specific causes of this disorder are complex and understanding the risk factors promptly is essential for prompt treatment and providing necessary support. The multifaceted nature of risk variables for PPDD encompasses various dimensions, including mental, physical, social, and lifestyle-associated aspects^1^. The key factors related to the risk of PPDD are slightly variable, presenting a formidable challenge in prediction and action. Biological variables, such as variations in hormones, exhibit complex interactions with psychological elements, including a prior history of disorders related to mental health, familial support, and individual resilience, in influencing the probability of PPDD onset. The recognition of these diverse influences necessitates the adoption of a holistic and coordinated approach to the determination of risk factors^2^.

PPDD is a deleterious medical condition that impacts around 12.8% of women who have recently given birth. Despite negative impacts on the health of mothers and their offspring, numerous women fail to be supplied with adequate care. The cost-effectiveness of preventative measures is observed in high-risk women; however, the current state of identifying such individuals is inaccurate. PPDD is a medical condition that can have profound and detrimental impacts on maternal well-being and their offspring’s development. Mothers may encounter enduring uncertainties regarding their capacity to provide adequate care for their children, challenges forming a strong emotional connection with them, and may also experience ideations related to causing harm to the child^3^.

Traditional statistical methods enable researchers to estimate risks by sequentially analyzing the associations primarily between two variables while frequently accounting for the influence of other variables. Moreover, machine learning techniques allow researchers to iteratively and concurrently examine numerous interconnected relationships among variables18. Additionally, these methods facilitate the development of data-driven predictive models, which can be assessed by quantifying performance metrics across all models to identify the most optimal predictive model. Machine learning (ML) can analyze intricate non-linear associations and combine and consolidate diverse data types from multiple origins. There has been a consistent rise in utilizing ML techniques in the medical field^4^.

This trend has had discernible impacts across various domains, such as oncology, cardiology, haematology, critical care, and psychiatry. Significantly, PPDD presents a distinctive scenario wherein a moderately elevated probability of developing a severe psychiatric disorder is accompanied by a highly accurate forecast of the specific timeframe within which these symptoms are anticipated to manifest. Given the significant societal burden of PPDD, utilizing ML for risk classification holds potential in an optimal scenario, offering considerable societal advantages. Given the annual birth rate of approximately 120,000 in Sweden, coupled with the fact that 14% of women experience PPD and exhibit various adjustments following childbirth, it is impractical to effectively monitor the entire population for early signs of depression after giving birth^5^. On the other hand, implementing regular and thorough monitoring of high-risk populations through postpartum assessments led by midwives or nurses can significantly enhance the provision of personalized and cost-effective mental healthcare services for mothers during the maternity period.

### Problems statement

PPDD is a mood disorder that is both pervasive and debilitating, significantly impacting a considerable proportion of women in the postnatal period. Despite its significant impact on mothers' mental well-being, PPDD remains insufficiently comprehended and frequently remains undetected until it progresses to a more severe state. Insufficient prompt treatment can exacerbate the illness, resulting in significant psychological anguish, limitation of the mother–child connection, and potentially enduring adverse effects on the child's growth. Identifying and addressing potential hazards linked with PPDD is highly challenging due to the intricate interplay between behavioural and naturally occurring factors and daily life choices. The intricate interaction of these factors makes it difficult to pinpoint those postpartum women particularly susceptible to PPDD. The absence of precise and fast detection methods places significant responsibilities on healthcare institutions and, most significantly, deprives women of the immediate support they desperately need^6^.

Traditional methods for evaluating the PPDD threat often rely on survey responses and clinical trial examinations. On the other hand, some of these techniques are prone to errors and might not have the sensitivity required to detect early symptoms of the medical condition. Establishing a broad, evidence-based, and responsive method for assessing risk variables cannot be emphasized sufficiently. This research addresses the pressing issue by utilizing advanced deep learning methods to create a forecasting framework to detect prospective threat variables accurately correlated to female PPDD.

### Motivation of the work

The motivation for carrying out this research arises from the urgent need to address the pervasive issue of PPDD, which significantly impacts women's mental health during the postpartum period and has extensive implications for their partners and their kids. The precise diagnosis of PPDD is frequently lacking, leading to unnecessary distress and challenges in forming a strong maternal-child bond. It is crucial to promptly identify detection techniques because of the complex interplay between mental disorders, biological, relational, and lifestyle factors and health uncertainties^7^. It requires a method that is based on data and covers all aspects. Applying state-of-the-art deep learning techniques, such as transfer learning methods and advanced deep learning models, offers a promising approach to improving the accuracy of identifying risk factors. The main aim of this study is to examine the lasting consequences of PPDD by implementing timely intervention and offering assistance. The objective is to reduce the burden on medical attention systems and support the overall mental well-being of the mother^8^.

### Contribution to the work

This research makes a significant contribution by introducing and implementing a new methodology for the timely identification and comprehension of potential risk factors associated with PPDD in women. The proposed method combines Convolutional Neural Network (CNN)-based transfer learning and an improved Bi-Directional Long Short-Term Memory (Bi-LSTM) model with an attention mechanism. The following is a comprehensive analysis of the particular contributions:

- **Integrated Hybrid Model:** The present investigation presents a novel integrated hybrid model which brings together the advantages of two separate deep learning architectures: (a) CNN-based transfer learning for textual and audio data separately and an improved Bi-LSTM framework incorporating an attention procedure to serve textual and audio sequential data combinable. This combined model provides an in-depth analysis of PPDD-associated risk factors by systematically considering a combination of image and textual data, which are critical for comprehending the multifaceted characteristics of PPDD.
- **Improved PPDD Risk Factor Identification:** Incorporating a Bi-LSTM model and an attention method significantly improves capturing intricate relationships between time and sequential trends within textual data. This enhancement leads to a more accurate and thorough evaluation of the mental and lifestyle variables crucial for predicting the risk of postpartum depression.
- **Improved data Feature Extraction:** The employment of transfer learning within the CNN module enables the extraction of important characteristics from the dataset. These features encompass various visual indicators linked to PPDD risk assessment, such as expressions on the face, non-verbal signals, and additional relevant visual clues. Transfer learning enables the model to take advantage of the knowledge acquired from pre-existing models, leading to improved precision in the extraction of features.
- **Advancement in Maternal Mental Health Research:** The combined framework conducts a thorough risk evaluation by examining risk factors from various data types and synthesizing these findings. The model improves the prediction accuracy of PPDD by incorporating data collected from the CNN, as mentioned earlier, along with Bi-LSTM components, which allows for a more comprehensive analysis of the interaction between different risk factors.
- **Improved performance Measuring parameter Results:** Integrating various factors enables a comprehensive examination of risk factors associated with PPDD. An experimental analysis is carried out on the PPDD online dataset (text with Audio records) collected from the public UCI dataset. Data pre-processing is applied to maintain the data integrity and achieve reliable model performance. In the evaluation of a test dataset, the proposed model demonstrates favourable performance in terms of better precision, recall, accuracy, and F1-score as compared to various existing deep learning models, including VGG-16^1^, CNN^9^, and CNN-LSTM^10^; these metrics serve as indicators of the model’s ability to differentiate among women who might be at risk of PPDD versus Normal. In addition, the feature importance analysis demonstrates that specific risk factors substantially impact the prediction of PPDD.

### Structure of the article

The complete paper is arranged in the following manner: it commences with a concise introduction that defines the problem and elucidates its significance, followed by a comprehensive examination of the relevant scientific literature. The section devoted to material and methodology comprehensively describes the data collection process, including the steps taken to gather and pre-process the data. Additionally, it outlines the innovative hybrid model that was developed, combining CNN-based transfer learning with an improved Bi-LSTM architecture incorporating an attention mechanism. The article subsequently provides a comprehensive overview of the dataset, experimental configuration, and outcomes, encompassing the experimental findings. An exhaustive analysis entails interpreting the findings, acknowledging any constraints, and proposing potential avenues for future research. The conclusion serves to recapitulate the main discoveries and their corresponding ramifications succinctly.

## Literature review

Postpartum depression is a significant mental health disorder that impacts brain function, behavioural patterns, and physical well-being. Individuals experiencing depression often encounter persistent feelings of sadness and hopelessness that can significantly disrupt their daily functioning. Some individuals may experience a lack of emotional attachment towards their infant, perceiving themselves as not being the biological mother or lacking feelings of love and concern for the child. The intensity of these emotions can range from mild to severe. The field of human–computer interaction is currently emphasizing the research field of emotion-aware computations^11^. This field typically focuses on techniques involving facial expression detection, speech interpretation, and the motion of an individual's assessment. Another significant area of research focuses on endowing computers with the capability to perceive human well-being, specifically methods for comprehending an individual’s emotions. Postpartum depression analysis is the focal point of extensive previous studies. Various studies have examined diverse treatment modalities employing machine learning and deep learning techniques, while others have focused on exploring the prevalence and risk factors associated with PPDD.

A Knowledge-Based Recommender System has been developed in^4^ to incorporate a comprehensive framework for tracking emotional well-being. This system utilizes an algorithm based on deep learning and employs a sentiment metric called ‘eSM2’. Currently, The system utilizes machine learning and deep learning methods to identify sentences containing negative content. The proposed approach employs a combination of CNN and Bi-LSTM algorithms to accurately detect individuals exhibiting symptoms of depression and stress, achieving respective accuracies of 0.87% and 0.91%. Moreover, this monitoring system can transmit alert messages to individuals who exhibit symptoms of depression or stress. Multiple studies employ publicly available information from websites such as Twitter, Instagram, and Facebook to examine the behaviour of patients on these forms of social media platforms and services^12^.

Examining social networking posts allows for identifying behavioural indicators associated with psychological illnesses such as depression, stress, and other disorders discussed in^5^. The strategy utilized in this study involves implementing the Co-training method, an instance of a semi-supervised machine learning method. This technique incorporates the discriminative capabilities of commonly employed classifiers, namely Random Forest (RF), Support Vector Machine (SVM), and Naive Bayes (NB). The resulting accuracy achieved by this method is 0.72 for RF and 0.83 for SVM and NB. A proposed solution involves utilizing a hierarchical post representation model (HPRM), the ‘MGL-CNN’ model, which aims to identify individuals with depression on social networking services. This approach encompasses both post-level activities and user-level activities. An alternative depression identification model known as SGL-CNN has been developed by modifying the total number of gates constituting the model's construction. The model presented in this study provides predictions for Precision, Recall, and F1-score values^13^.

The study^14^ primarily uses natural language processing and text categorization to identify depression. The system primarily identifies lexicon terms frequently observed in individuals diagnosed with depression. This study's findings enhance the accuracy and performance of the model. The most notable aspect of the study is the utilization of Bigram analysis in conjunction with a SVM classifier, which effectively aids in the identification of depression with a commendable accuracy rate of 82.32% and an F1-score of 84.27%. Certain studies employ DCNN and ANN in the identification and classification of depression. Two models, the Deep and Shallow models, have been proposed to analyze depressive symptoms. This paper presents a novel approach that integrates text and video features by leveraging deep and shallow algorithms. The proposed methodology encompasses utilizing an RF method to classify depression based on the estimation of scores. It has been suggested that a new method could be used to detect depression, which uses linguistic signals and extracts content from responses from individuals based on the language they use^15^.

The suggested approach^16^ primarily advances the field of mental health detection. It incorporates a Bi-LSTM framework with an attention layer to analyze textual information, a 1-D CNN to analyze Audio signals, and a fully interconnected system that combines the outcomes of both prior models to evaluate the seriousness of depressive states. In the preceding study, text data obtained from university students is utilized to identify symptoms of depression within the same population. The DISVM method categorizes data acquired compared to an input source, ultimately identifying depressive disorders as psychological disorders. Based on the findings reported in research^17^, the precision values obtained for DISVM were 0.814% for the training set and 0.826% for the testing set. Several research studies employ multi-modal information, including audio, video, and text, to predict patients' mental health conditions. The output is categorized into distinct levels of depression to account for the varying degrees of severity experienced by the patient.

Various approaches^19,20^are employed for the identification of depression and the categorization of textual, visual, and auditory characteristics. Based on the findings reported in the article^10^, the f1-score is determined to be 0.84%, while the precision is measured at 0.82%. Certain algorithms accurately forecast the clinical signs and extent of depression, whereas other approaches do not yield satisfactory outcomes. Based on the literature mentioned earlier, researchers have concluded that scientists have proposed numerous solutions to address the issue of detecting depression. The escalating prevalence of depression has prompted numerous proposed solutions; however, these interventions have not yet demonstrated a high level of accuracy, resulting in significant losses. Certain researchers utilize data from social media sites, the accuracy of which may vary^22^.

The utilization of online communities^23^ that include Facebook and Twitter to predict depression carries the potential of erroneously identifying symptoms of depression. It will be a mistake if symptoms seen on social media sites that are accessible online are incorrectly anticipated. If the outcomes achieved are not precise, it becomes difficult to assess the specific risk of depressive disorders. On occasion, individuals on social networking sites may purposefully or inadvertently share narratives that convey feelings of depression or sadness, thereby potentially influencing the overall efficacy of depression detection systems. Hence, researchers cannot comprehensively rely on social media sites^24^.

The researchers commonly employ reliable databases when constructing a depression identification system. Investigators require an automated depression detection system that attains high precision and minimizes losses as evaluated by the system. Combining the characteristics of audio recording samples, video specimens, and textual responses can precisely determine outcomes. Using a Deep Neural Network enables the system to forecast depression with relative ease accurately. Developing a depression recognition system is possible once the model has been trained and comprehensively understands the various features in multimedia, text, and audio data. Table 1 presents a comprehensive analysis of existing PPD research.

**Table 1:** Comprehensive analysis of existing PPDD research.

References	Technique	PPDD data source	Key findings	Limitations
^15^	CNN and LSTM	Medical Intuitions and Survey	High-precision identification of key factors associated with risk; a holistic strategy integrating textual and visual data.	Limitations in the number of samples, data quality, and generalization ability.
^16^	CNN Model	E-Health Dataset	Several risk factors were determined, which include familial history and anxieties.	The analysis is restricted to textual data and is based on a limited dataset.
^17^	Hybrid Machine Learning models SVM and Logistic Regression	Online Social Media	Emphasized the significance of facial expressions through the risk of postpartum depression.	Restricted to sleep-related uncertainty.
^18^	Time Series Analysis	University of Texas Dataset	Discovered many risk factors through text and image statistics.	Detection rate is poor.
^19^	Genetic Algorithm	Diverse Medical care dataset	Found PPDD-prone genetic markers.	Limited to genetics; ethical concerns.
^20^	Logistic Regression	Twitter dataset	Discovered partner support along with self-care crucial to PPDD prevention.	Qualitative nature limits generalizability.
^21^	Natural Language Processing	Online Social Media	Sleepiness detected by smart devices relates to PPD risk.	Work only on textual datasets.
^10^	Time Series Analysis	Patient Survey	Reviewed postpartum depression discussions, focusing on support from peers.	The model does not lend itself to being interpreted.
Proposed Model	CNN-based TL and Improved Bi-LSTM with Attention Method	PPDD UCI (Text with Audio) dataset	Discovered many risk factors with High accuracy through text and audio statistics.	Data privacy ethics.

## Materials and methods

This section provides an overview of the dataset and techniques employed in the investigation, which focused on identifying risk factors for depression after childbirth in women. The subsections cover a range of topics, such as PPDD dataset standards, data preliminary processing approaches, feature selection and extraction techniques, the operation of the proposed hybrid framework, and important parameters for performing consequence comparisons.

### Dataset

The proposed hybrid method entails the utilization of transfer learning based on two CNN architectures with an improved Bi-directional Long Short-Term Memory model and attention method. The main goal of this integrated method is to improve the accuracy and thoroughness of understanding concerning risk variables linked to PPDD, consequently strengthening the promptness and efficiency of prompt identification and action approaches for women during the postpartum phase. To assess the robustness of the proposed approach, this investigation employs a widely recognized text-mixed audio PPDD dataset called "PPDD". The repository is obtained from the publicly available UCI dataset^24^. The dataset comprises two directories of patient records for individuals diagnosed with PPDD. Table 2 presents the analysis of risk variables that plays an important role in determining the PPDD patients.

**Table 2:** Analysis of risk variables that play an important role in determining the PPDD patients.

Attribute	Key domain	Risk factor	Sample
General Factors (GFs)	The social demographic information	Age	18–24, 25–30, 30–35, 40–45, 45–50, and above 50
		Income Type	Poor, Rich, Moderate
		Education	School, College, None
Patient Health Questionnaire-9 (PHD-9) related to pregnancy	Pregnancy Features	Depression history	Inadequate weight, development
		Challenges with Infertility	Less birth weight
		Pregnancy thoughts Maternal	Highly satisfied in some aspects but not in others
		Pregnancy thoughts Paternal	Highly satisfied in some aspects but not in others
Edinburgh Postnatal Depression Scale (EPDS) Mother	Maternal Features	Offspring count	Single, Two, More
		Marital status	Unmarried, Married
		Hospital Distance	Less than 5, more than 5
Postpartum Depression Screening Scale (PDSS)	Obstetric	Relationship with parents	Close, Not Close, None
		Delivery Mode	Yes, No
		Labour Induction	Yes, No
Edinburgh Postnatal Depression Scale (EPDS) Child	Child Features	Weight at birthtime	Normal, Over, Less
		Birth week	Normal, Over, Less
Patient Health Questionnaire-9 (PHD-9) related to workplace	Daily life challenges	Workplace stress level	Yes, No, None
		Being worried about returning to work	Yes, No, None

#### Textual features

In the PPDD dataset the first folder contains 1550 textual records in the dataset. The Table 2 presents the risk variable assessment of textual features that play an important role in determining the PPDD patients.

- **Attributes:** ‘Age’, ‘Trouble sleeping at night’, ‘Irritable towards baby and partner’, 'Problems concentrating and making decisions’, ‘Overeating and loss of appetite’, ‘Feeling of guilt’, ‘Problems of bonding with baby’, ‘Suicide attempt’, ‘Feeling sad and tearful’.
- **Targeting attributes:** ‘Feeling anxious’.

**Figure 1:**
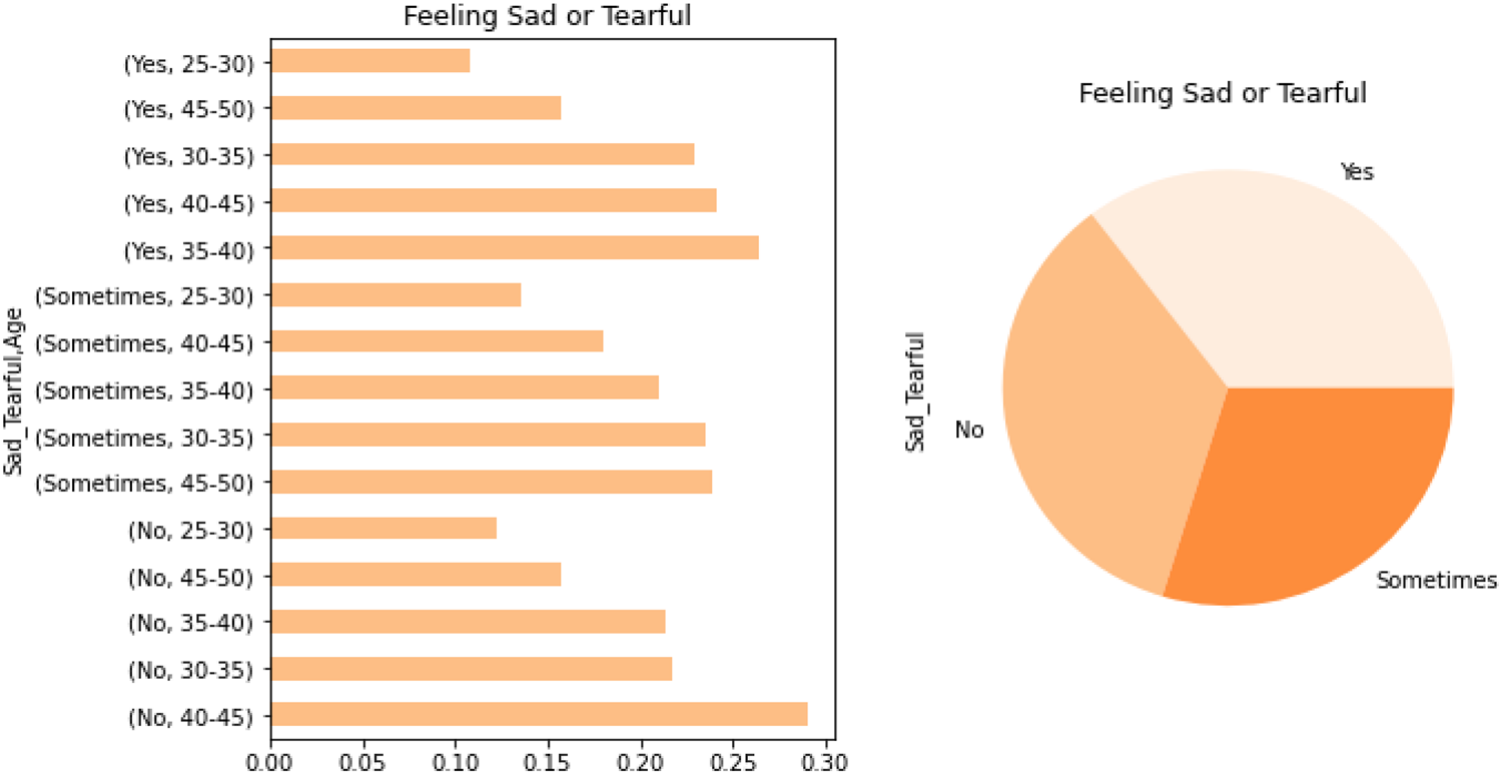
PPDD dataset textual feature representations for Feeling Sad or Tearful.

**Figure 2:**
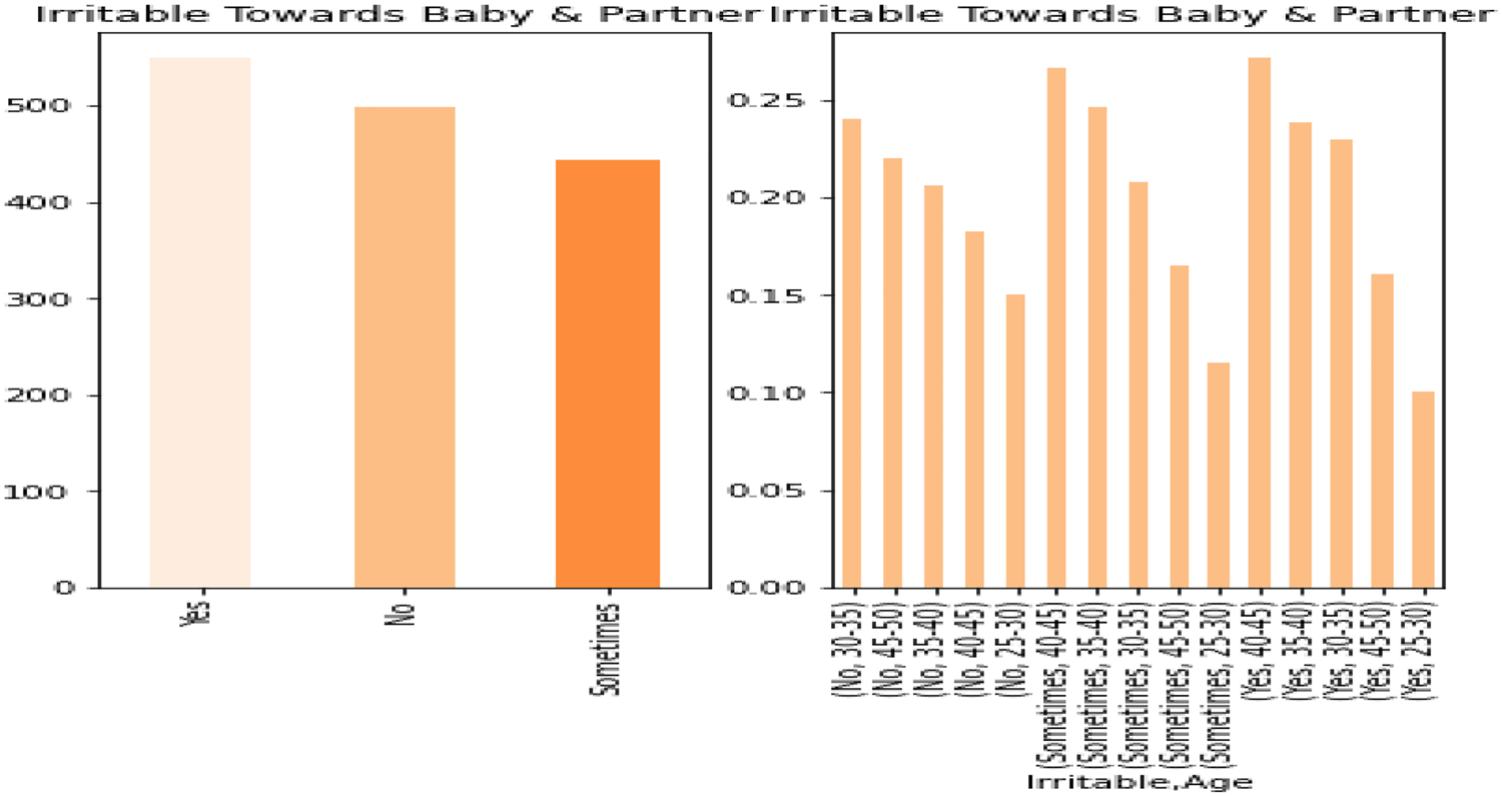
PPDD dataset textual feature representations for Irritable baby and partner.

Figure 1 presents the visual of the PPDD dataset textual feature representations (Feeling Sad or Tearful), and Fig. 2 presents the PPDD dataset textual feature representations (Irritable towards baby and partner).

#### Audio contents

**Figure 3:**
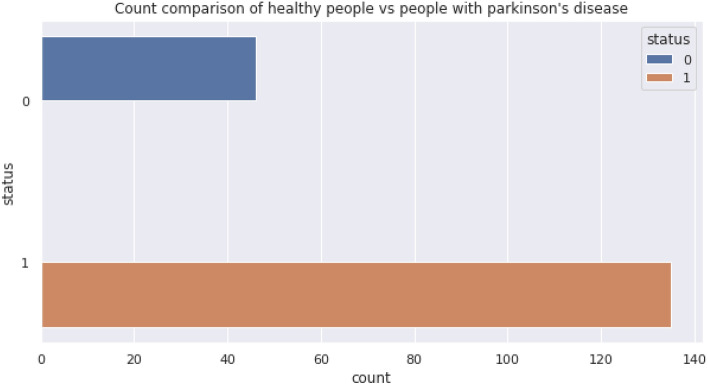
Total count of healthy vs. infected PPDD women in the dataset.

**Figure 4:**
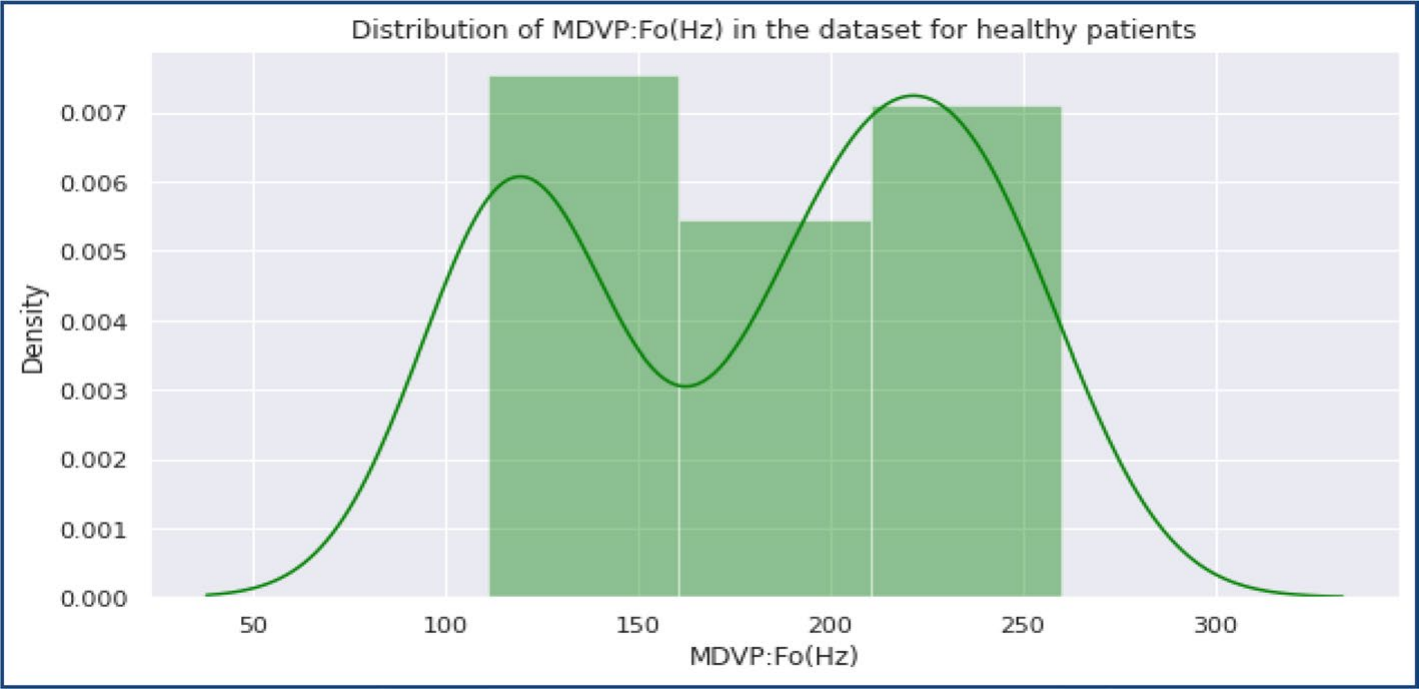
Distribution of MDVP-Fo in the dataset for Healthy Women set.

**Figure 5:**
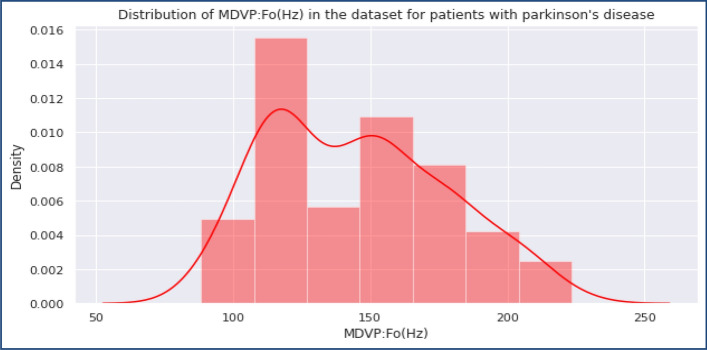
Distribution of MDVP-Fo in the dataset for Unhealthy Women.

In the PPDD dataset, the second folder contains 195 speech records^25^. Figure 3 presents the total count of healthy vs. infected PPDD woman in the dataset, and Figs. 4 and 5 presents the Distribution of MDVP-Fo in the dataset for Healthy and Unhealthy Women.

### Data pre-processing

Pre-processing the PPDD dataset for detecting risk factors associated with postpartum depression, which involves integrating text and audio data, encompasses multiple stages to prepare the PPDD final dataset. The audio dataset is converted from audio to spectrogram images by employing the “librosa.feature.melspectrogram” function. The picture produced as a result represents the varying frequency components of the audio signal as it changes over time^26,27^.

#### Data cleaning and handling

Removing redundant entries in datasets is necessary to ensure data integrity. Additionally, the issue of missing data can be resolved by eliminating samples that contain incomplete information. Finally it is important to address inconsistencies in the data and detect outliers in textual datasets^28^.

#### Feature extraction

**Figure 6:**
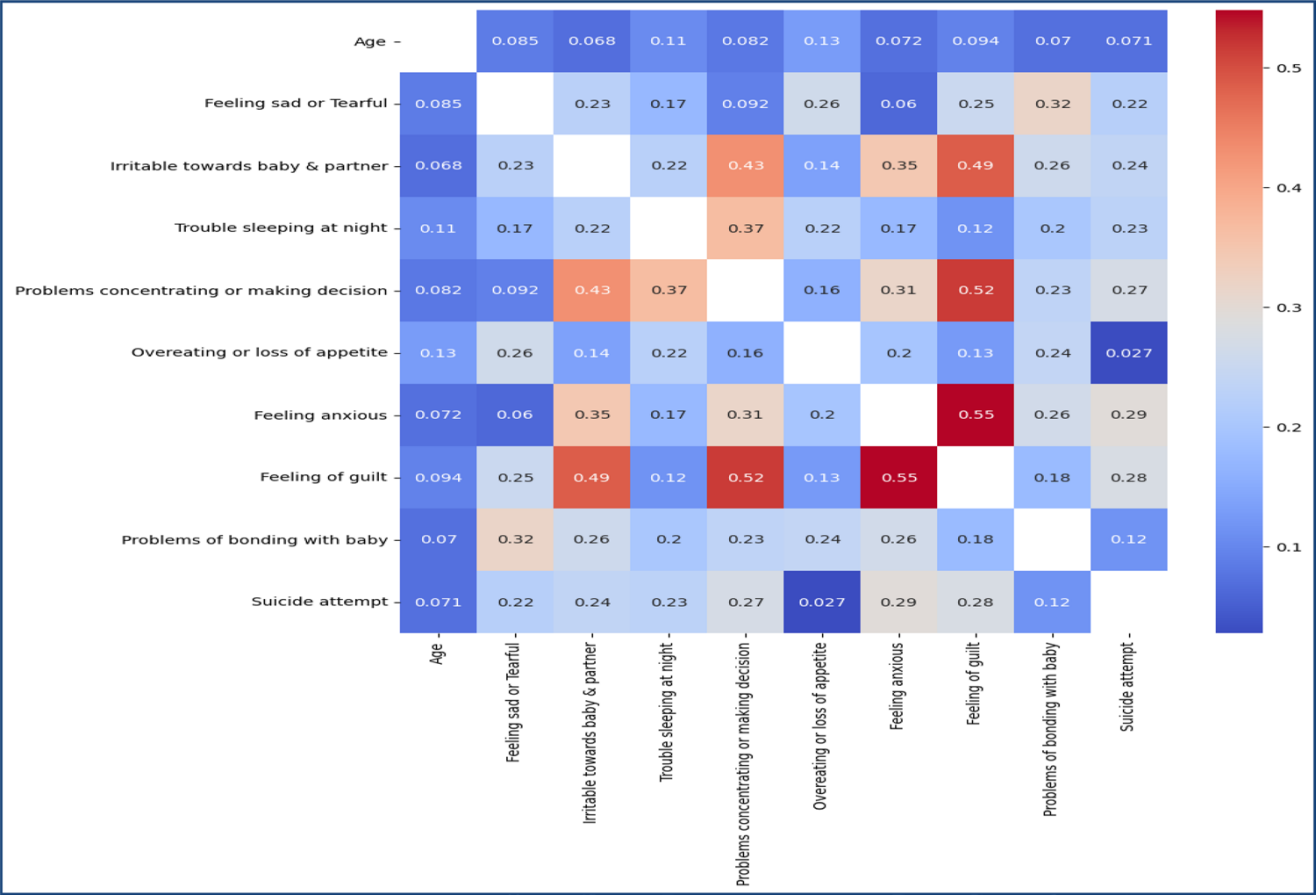
Feature co-relationship of PPDD textual attributes.

**Figure 7:**
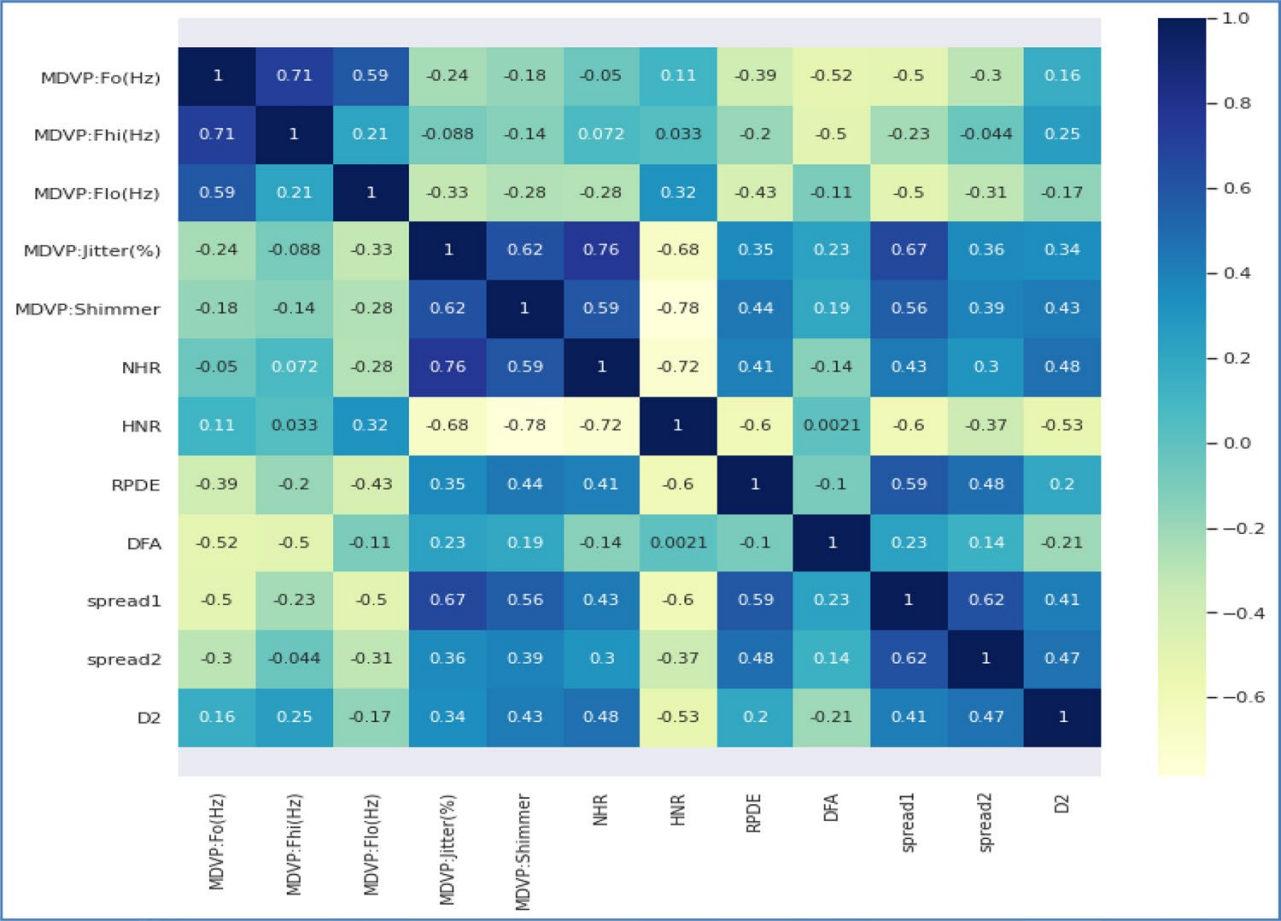
Feature co-relationship of PPDD Audio data attributes.

The proposed hybrid strategy for PPDD-associated risk factors detection emphasizes the significance of feature extraction as a crucial step in converting unprocessed data into significant representations^29^. Textual data in the medical field often goes through pre-processing procedures, including tokenization, converting all text to lowercase, eliminating stop words, and transforming the text into numerical vectors. The tokenized and vectorized text is subsequently processed via an embedding layer to acquire compact vector representations for individual words. The subsequent step involves utilizing a Bi-LSTM layer that incorporates a method of attention. This layer effectively captures the clinical notes’ temporal relationships and contextual information. When dealing with audio data, extracting and normalizing specific characteristics, including Mel-frequency cepstral coefficients (MFCCs)^30^ and spectrograms, is common practice. Figure 6 presents the feature co-relationship of textual features, and Fig. 7 presents the feature co-relationship of audio features.

**Figure 8:**
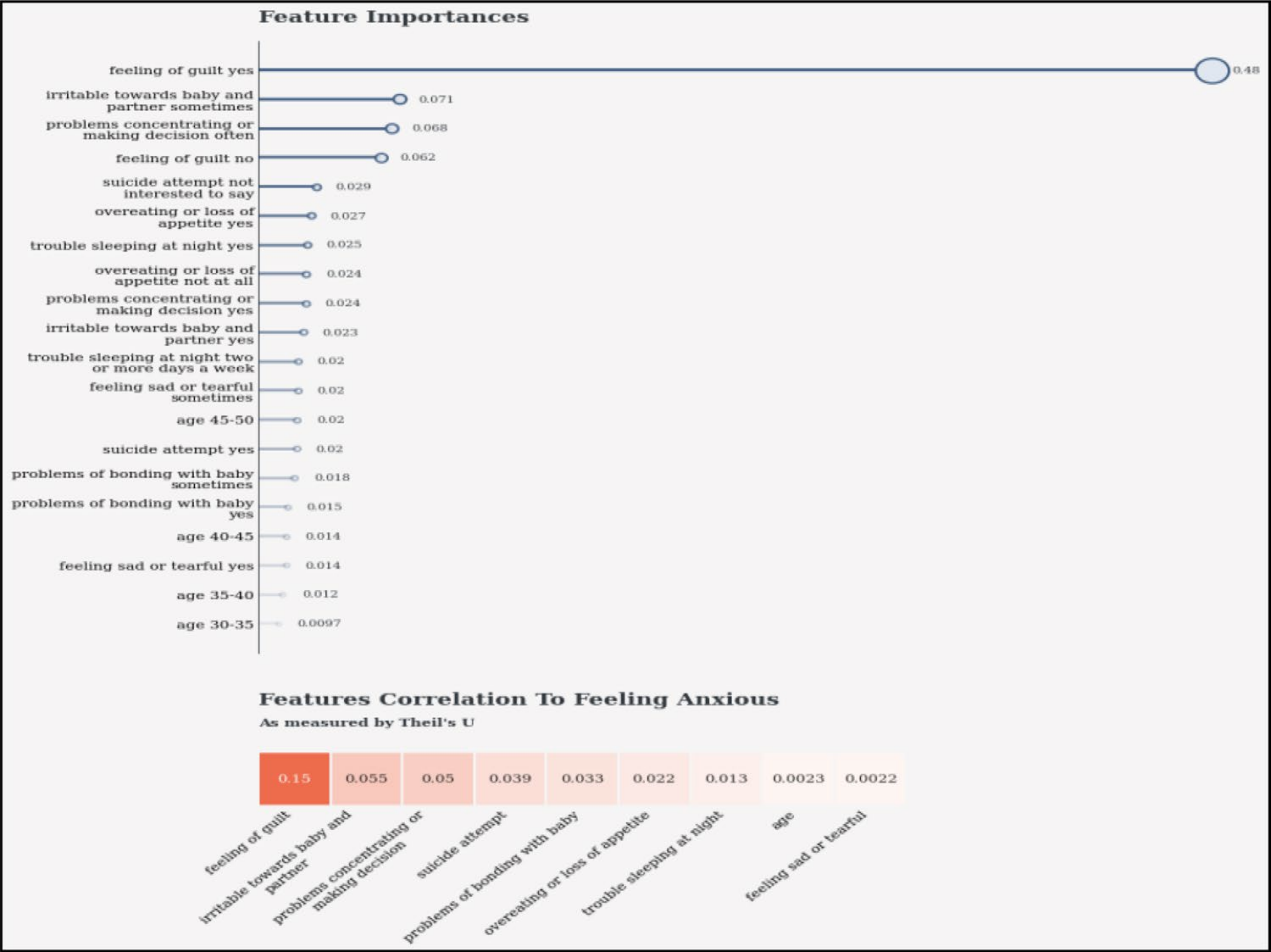
Feature importance of the PPDD dataset in terms of feature co-relation with feeling anxious.

A trained CNN structure (depending on Transfer Learning) can interpret the audio characteristics into an image-like structure. The CNN model is designed to extract and represent high-level features that effectively record spatial patterns within the audio data. Combining characteristics from both modalities is achieved by employing concatenation or fusion methods, resulting in an extensive audio and text-based data summary. The combined feature representation plays a fundamental role in the model's forecasting tasks by offering a comprehensive perspective for identifying risk factors associated with PPDD^31^. Figure 8 presents the feature importance analysis of the PPDD dataset in terms of feature co-relation with feeling anxious.

### Proposed framework for PPDD Detection

**Figure 9:**
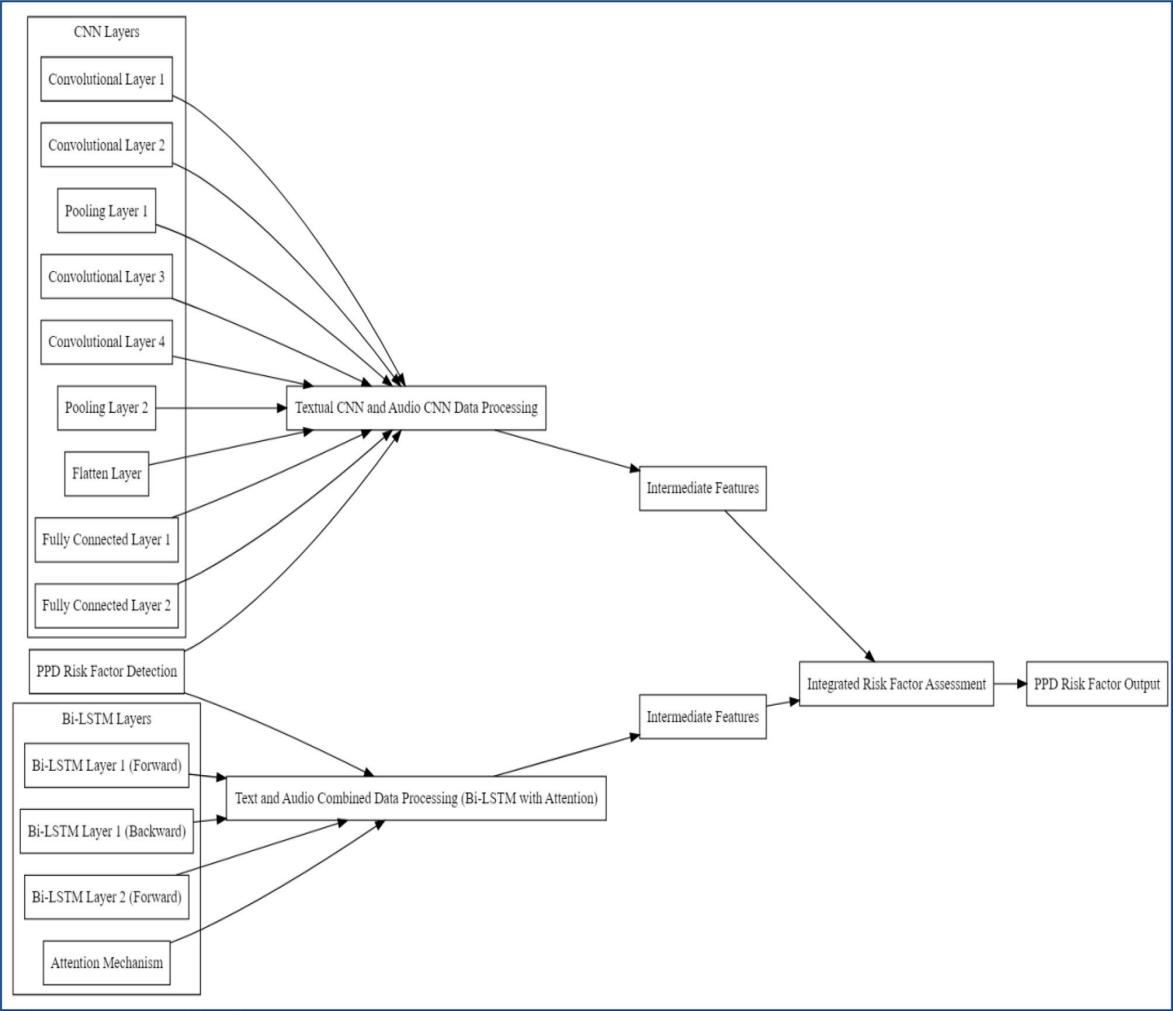
Architecture of proposed model.

The proposed model for detecting risk factors of postpartum depression disease is an advanced hybrid system that integrates transfer learning using CNN with an improved Bi-LSTM model that incorporates a method of attention. The objective of this model is to utilize the abundant information contained within audio and textual information to offer a comprehensive evaluation of risk factors associated with PPDD. The key modules of a proposed model include CNN structure with transfer learning improved Bi-LSTM with Attention method^32^. Figure 9 presents the architecture of the proposed model for PPDD detection in women.

#### Proposed model functioning

**Figure 10:**
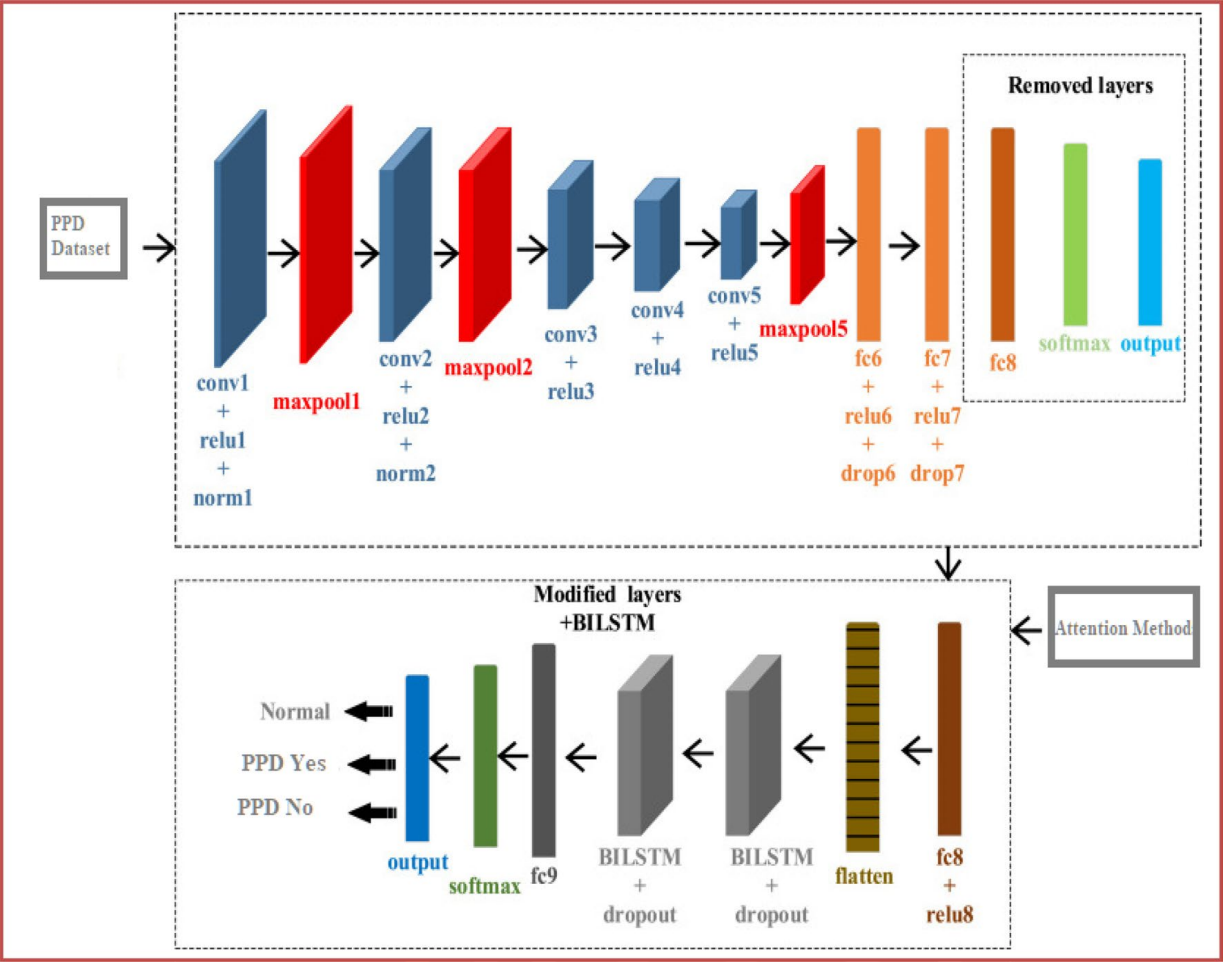
Working of proposed model.

The structure of the proposed model consists of three parts. The first part is a textual CNN structure that exclusively employs text characteristics. This model is trained exclusively on text features. The second part is an audio CNN model that exclusively employs audio features such as Mel spectrograms and MFCCs. This model is trained solely on audio features. The third and final part is a hybrid model combining audio and textual models^33^. The structure comprises several layers, including the ‘data input layer’, ‘convolutions layer’, ‘pooling layer’, ‘fully linked layer’, ‘SoftMax layer’, and ‘outcome layer’. The process by which these layers begin to connect is known as neural network architecture. Furthermore, additional layers, including the ‘dropout layer’, ‘activation function’, ‘flatten layer', and ‘batch normalization’, were used in the CNN structure. Figure 10 presents the complete work of the proposed hybrid model.

The proposed hybrid model utilizes audio features, including MFCC, COVAREP, and Mel Spectrograms. Pre-trained models, which already have characteristics of PPDD patients’ content outcomes, are used for text characteristics. Mel Spectrograms produce the most optimal outcomes among all the features used for the audio CNN approach. The proposed model utilizes a combination of audio and text features processed through a CNN and Bi-LSTM architecture. The output of this process is a set of binary labels which show depressed or Non-depressed. The proposed system is utilized for the detection of PPDD. It autonomously predicts how much a woman is experiencing depression or not after childbirth. The proposed model contains the following key layers^34^.

- **Convolution Layer:** The initial or primary layer holds the utmost significance within a neural network. This layer creates the foundation of a complete neural network. The primary function of the possible Convolution layer remains to identify the nature of the input, such as text characteristics, audio characteristics, or a combination of both. The entered image is convolved along with a filter of the specified size within the convolution layer, and the resulting image is generated. A feature map is generated in the results. Convolution layers utilize filter kernels, which at first are weight matrices. These weights are modified by implementing the backpropagation technique in the convolution layer.
- **Max-Pooling Layer**: Between two convolution layers is the pooling layer. The main function of a mixing layer is to reduce the dimensions of the input. This layer facilitates the model in diminishing the information's current size and refreshing it with only the essential facts. This layer enhances the effectiveness of a neural network. It mitigates undesirable attributes in the data. This layer is an intermediary between the convolutions and fully linked layers^35^. Pooling layers aid in mitigating overfitting issues that can occur in neural networks.
- **Activation Function:** We utilize the ReLU activation function. The Rectified Linear Unit (ReLu^36^ is a mathematical function that represents non-linearity. This layer substitutes any negative information alongside zero-value pairs. The significance of this layer lies in its ability to determine which information should be transmitted to the subsequent convolution layer and which information should be disregarded.
- **Batch Normalization Layer:** Batch Normalization layers standardize the output of preceding layers. It facilitates the modelling process by enabling the efficient acquisition of features. It enhances the stability and accelerates the performance of the model. It enhances the speed of processing and learning of the model.
- **Dropout layer:** The dropout layer is utilized to mitigate overfitting in the model. It stochastically omits certain values in the neural network, resulting in an accelerated learning rate for the model. The dropout layer is positioned following the fully connected layer. The optimal value with dropout is 0.25. It can result in sluggish training within neural networks.
- **Fully linked Layer:** A Completely linked layer is typically positioned before the output sorting layer. The classification process commences within this layer, and the outcomes automatically update. When constructing a neural network, it is crucial to incorporate one or two fully connected layers.

#### CNN-based transfer learning

**Figure 11:**
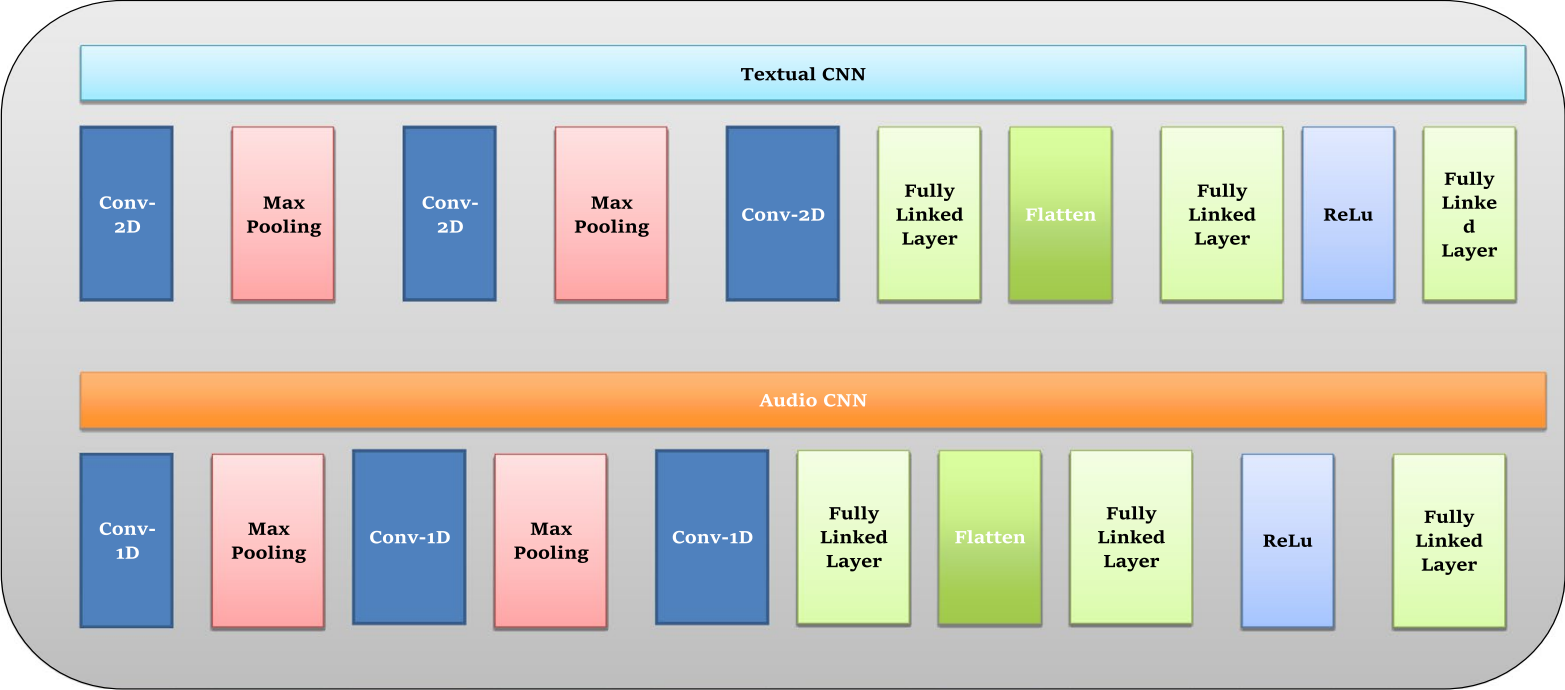
The text and audio CNN layers in the proposed model.

The CNN section operates as the text and audio (visual) analysis processing module and can process text and audio data. Transfer learning is utilized in our study, which involves using a pre-trained CNN model, specifically AlexNet. This model is further refined using our own PPDD dataset. The convolutional neural network executes convolution and pooling procedures, thereby acquiring hierarchical characteristics from the dataset^37^. By adjusting the weights of the CNN models (AlexNet) that were already trained with PPDD datasets, a transfer learning method modifies the models to our particular task. The process of fine-tuning allows the model to apply its learned patterns from large datasets, thereby recognizing significant visual characteristics indicative of the risk of PPDD. Figure 11 presents the text and audio CNN layers.

The CNN module incorporates transfer learning using the pre-trained AlexNet model, applying a series of layers of convolution and pooling to extract features. As mentioned earlier, the layers encapsulate a breadth of acquired knowledge derived from various PPDD datasets. These layers effectively capture visual characteristics of a sophisticated nature that are signs of the risk of PPDD. Convolutional along with fully connected layers are included in the AlexNet model’s exact number of sixteen layers.

#### Improved Bi-LSTM with attention method

**Figure 12:**
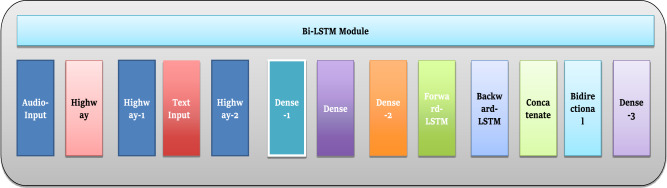
Layers in proposed model for Bi-LSTM module.

An important understanding of the mental and behavioural variables associated with PPDD potential is gained from the textual information, which includes survey outcomes and stories. To accurately represent the sequential dependencies and complex details in the textual information, we employ an improved Bi-LSTM model incorporating an attention procedure. The Bi-LSTM is a neural network with a recurrent structure designed to analyze textual content sequentially. It can consider the sequence and context of each word to make accurate predictions or classifications^38^. Figure 12 presents the number of layers in the proposed model for the Bi-LSTM module.

The Bi-directional characteristic of this phenomenon enables it to consider both preceding and subsequent words, a critical aspect in comprehending narratives. The inclusion of the attention mechanism enhances performance by assigning differential significance to distinct words within the input text. This functionality allows the model to focus on pivotal words and phrases throughout the text, which can probably serve as warning signs for PPDD^35^.

In addition to a graphical analysis from the CNN, the Bi-LSTM element enhances the proposed model's performance through its sequential processing ability. The two layers, including one forward and one backward layer, produce a bidirectional circulation of information that collectively makes up the Bi-LSTM building design. An attention procedure is integrated to augment the model's comprehension of textual data within its context. The attention mechanism comprises multiple layers responsible for distributing different levels of significance to each word within the textual input, thereby enhancing the analysis of narrative information with a nuanced approach.

#### Model integration and fusion

The main advancement of our methodology resides in the fusion of the outcomes from both the Convolutional Neural Network and Bidirectional Long Short-Term Memory models, resulting in the development of an extensive risk evaluation framework. The integration of audio (converted into its equivalent images) and text-based data in the framework allows for utilizing knowledge from both domains and considering the interrelatedness of risk variables in PPDD. The combined framework leverages the extracted features of the dataset obtained from the CNN and incorporates contextual knowledge from the textual information to generate predictions regarding risk factors.

The attention mechanism enhances this process by emphasizing the most useful words within the text. By integrating these perspectives, the model provides a sophisticated and all-encompassing evaluation of risk factors associated with PPDD, considering the intricate interaction between mental and lifestyle elements. The hybrid model we propose exhibits an extensive architecture comprising approximately 18 layers. This count includes the 16 layers in the Convolutional Neural Network and the additional 2 layers in the Bidirectional Long Short-Term Memory model, which incorporates an attention mechanism. Integrating these layers facilitates a comprehensive and precise evaluation of risk factors associated with PPDD by concurrently capturing both visual and textual indicators while considering their interrelatedness. Algorithm 1 presents the pseudo code of proposed hybrid TL-based CNN with Improved Bi-LSTM for PPDD analysis.

#### Algorithm for proposed model

Algorithm 1 presents the algorithm of the proposed hybrid model.

**Algorithm 1:**
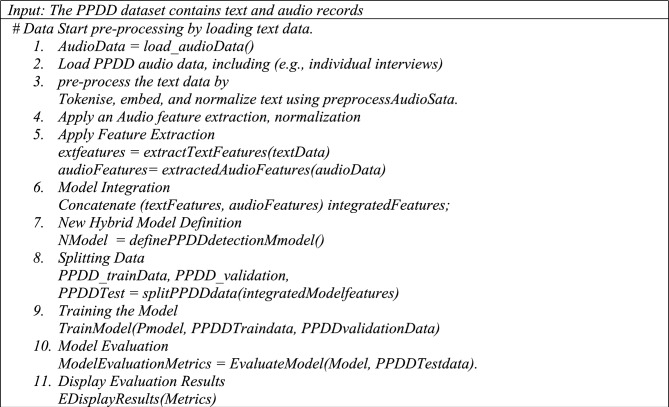
Hybrid TL-based CNN with Improved Bi-LSTM for PPDD analysis.

### Comparison parameters

The efficiency of the proposed hybrid model was measured using the following performance indicators. Equations (1–4) employed the subsequent representations. TP refers to true positives, which are instances correctly identified as positive. FP stands for false positives, which are instances incorrectly identified as positive. TN represents true negatives, which are instances correctly identified as negative. FN denotes false negatives incorrectly identified as negative^30^.

- **Confusion Matrix:** A table is used to evaluate the efficacy of a machine and deep learning algorithm by comparing the predicted labels with the actual labels in a given dataset.
- **Accuracy (Acc)**: It calculates the proportion of correctly classified incidents compared to the total number of occasions in data collection as describe by Eq. (1).
  1 $$Acc = \left\{ {\frac{{\left[ {TP + TN} \right]}}{{\left[ {TP + FP} \right] + \left[ {TN + FN} \right]}} } \right\}$$
- **Precision (Pre):** It is also called PPV “positive predictive values”. It is a metric utilized to evaluate the efficiency of a deep learning technique by calculating the ratio of correctly predicted positive incidents (true positives) to all predicted positive incidents as describe by Eq. (2).
  2 $${\text{Pre }} = \left\{ {\frac{{\left[ {TP} \right]}}{{\left[ {TP + FP} \right]}}} \right\}$$
- **Recall (Rec):** It is also called “Sensitivity” or “TPR (true positive rate)”. It is a metric used to assess the effectiveness of a deep learning method by determining the proportion of precisely anticipated positive events (true positives) out of all anticipated positive events as describe by Eq. (3).
  3 $${\text{Rec}} = \left\{ {\frac{{\left[ {TP} \right]}}{{\left[ {TP + FN} \right]}} } \right\}$$
- **F1-Score (F1S):** The F1 score balances precision and recall by calculating their harmonic mean as describe by Eq. (4).
  4 $${\text{F}}1{\text{S}} = \left\{ {2*\left[ { \frac{{\left( {Pre*Rec} \right)}}{{\left( {Pre + Rec} \right)}}} \right]} \right\}$$
- **Receiver Operating Characteristic (ROC):** The ROC curve illustrates the balance between sensitivity and specificity across various threshold values.

## Simulation results and comparison

The proposed and existing models, i.e., VGG-16, CNN, and CNN-LSTM, are implemented over the PPDD text and audio dataset collected from UCI.

### Configuration details

Data splitting is essential in deep learning and data analysis as the foundation for model development and evaluation. For this study, we have carefully partitioned our PPDD collection into three separate subsets: training (comprising 70% of the data), validation (comprising 15% of the data), and testing (comprising 15% of the data). The training set, which contains the largest portion of the data, serves as the fundamental basis for the learning process of a deep learning model. The validation set, which is deliberately reserved, plays a crucial role in optimizing the performance of our model. It functions as a point of evaluation, enabling us to optimize Hyperparameters, address overfitting, and choose the most effective model^32,34^. Finally, the test set, which is completely separate and not observed by the simulation over training, acts as the definitive evaluation. The hardware and software details are presented in Table 3.

**Table 3:** Hardware and software details.

Category	Key element	Details
Software	Operating System	Linux
	Python	Version 3.7 and above
	Deep Learning Libraries	PyTorch, Tensor Flow, Keras
	IDE	Integrated Development Environment
	Pre-Processing of data	Pandas and Numpy
	Visualization libraries	metaplot library
	Audio data pre-processing	Librosa
Hardware	Processor	I-7 or equivalent
	RAM	8 GB and Above
	GPU	Nvidia

The proposed hybrid model is based on two robust deep learning architectures: CNN and improved Bi-LSTM parameters. Details with values are presented in Table 4.

**Table 4:** CNN and Bi-LSTM parameter details.

Model	Layer	Output structure	Attribute	Activation Function	Any other details
CNN	Input layer	Batch size (1,128,128)	None	None	Text and audio input layer
	Conv-2D layer	Batch size, (32,126,126)	Filter 32, Size of Kernel (3,3)	ReLu Activation ()	Feature extraction
	Max Pooling-2D Layer	Batch size, (32,63,63)	pooling size (2,2)	None	Perform down sampling
	Conv-2d Layer	Batch size, (64,61,61)	Filter 64, Size of Kernel (3,3)	ReLu Activation ()	Additional layer
	Flatten- Layer	Batch size, (64,61,61)	None	None	Make output flatten for processing
LSTM	Input layer	Time steps, 128	None	None	Combine input (Text with Visual)
	LSTM layer	Time steps, 64	LSTM units, 64	tanh activation ()	Sequential data by LSTM
	Attention Layer	Time steps, 64	None	None	To enhance BLSTM
	Dense Layer	Time steps, None	None	SoftMax Activation ()	Output finalizing
Epoch	Simulation -1		training, testing and validation		100
	Simulation -2		training, testing and validation		140
Data Split	Simulation -1		training, testing and validation		70%, 15%, 15%
	Simulation -2		training, testing and validation		80%, 10%, 10%

## Experimental results

The experimental results are calculated for proposed hybrid and existing models, i.e., VGG-16^3^, CNN^1^, and CNN-LSTM^2^ over PPDD text and audio datasets^24^. Experimental results are calculated by applying two simulation scenarios.

### Simulation 1

In simulation 1, the dataset is divided into 70%, 15%, and 15% for training, testing and validation and the results were computed for 100 epochs for proposed and existing deep learning models.

Table 5 presents the simulation results, comparing the proposed and existing model for the PPDD dataset over 100 Epochs. The proposed model achieved an Accuracy of 96.125%, precision of 96.152%, F1-score of 96.710%, Recall of 96.471% and Mean Square Error of 0.01121. Similarly, we have implemented existing methods on the same dataset. The existing CNN model achieved an Accuracy of 88.279%, precision of 88.142%, F1-score of 88.817%, Recall of 88.724% and Mean Square Error of 0.0397. The existing VGG-16 model achieved an Accuracy of 89.027%, precision of 89.077%, F1-score of 89.074%, recall of 89.007% and Mean Square Error of 0.03402 and existing CNN-LSTM model achieved an accuracy of 91.071%, precision of 91.708%, F1-score of 91.015%, Recall of 90.307% and Mean Square Error of 0.02781. The above experimental results prove that the proposed model performed better than existing models.

**Table 5:** Results comparison of proposed and Existing model for PPDD dataset over 100 Epochs.

Technique	Accuracy %	Precision %	F1-score %	Recall %	Mean Square Error
Proposed Hybrid Model	96.125%	96.152%	96.710%	96.471%	0.01121
CNN Model	88.279%	88.142%	88.817%	88.724%	0.0397
VGG-16	89.027%	89.074%	89.007%	89.007%	0.03402
CNN-LSTM	91.071%	91.708%	91.015%	90.307%	0.02781

**Figure 13:**
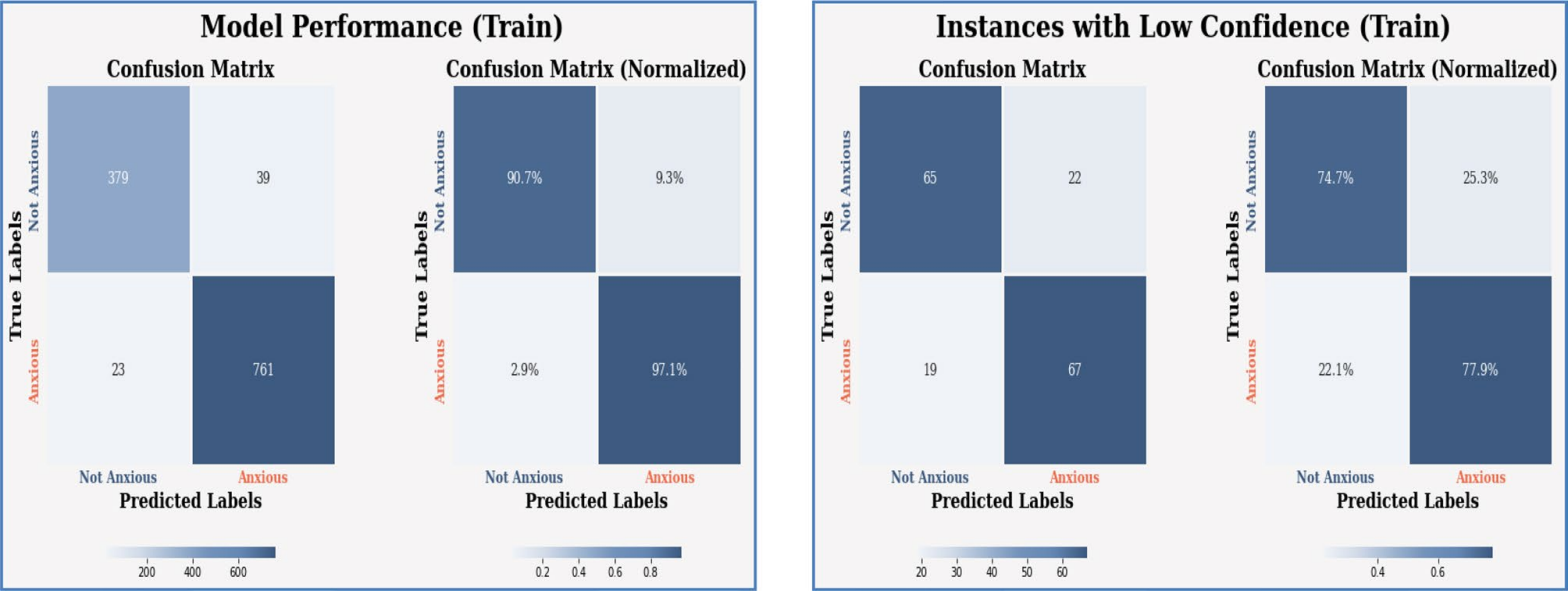
Confusion matrix results for the proposed model.

**Figure 14:**
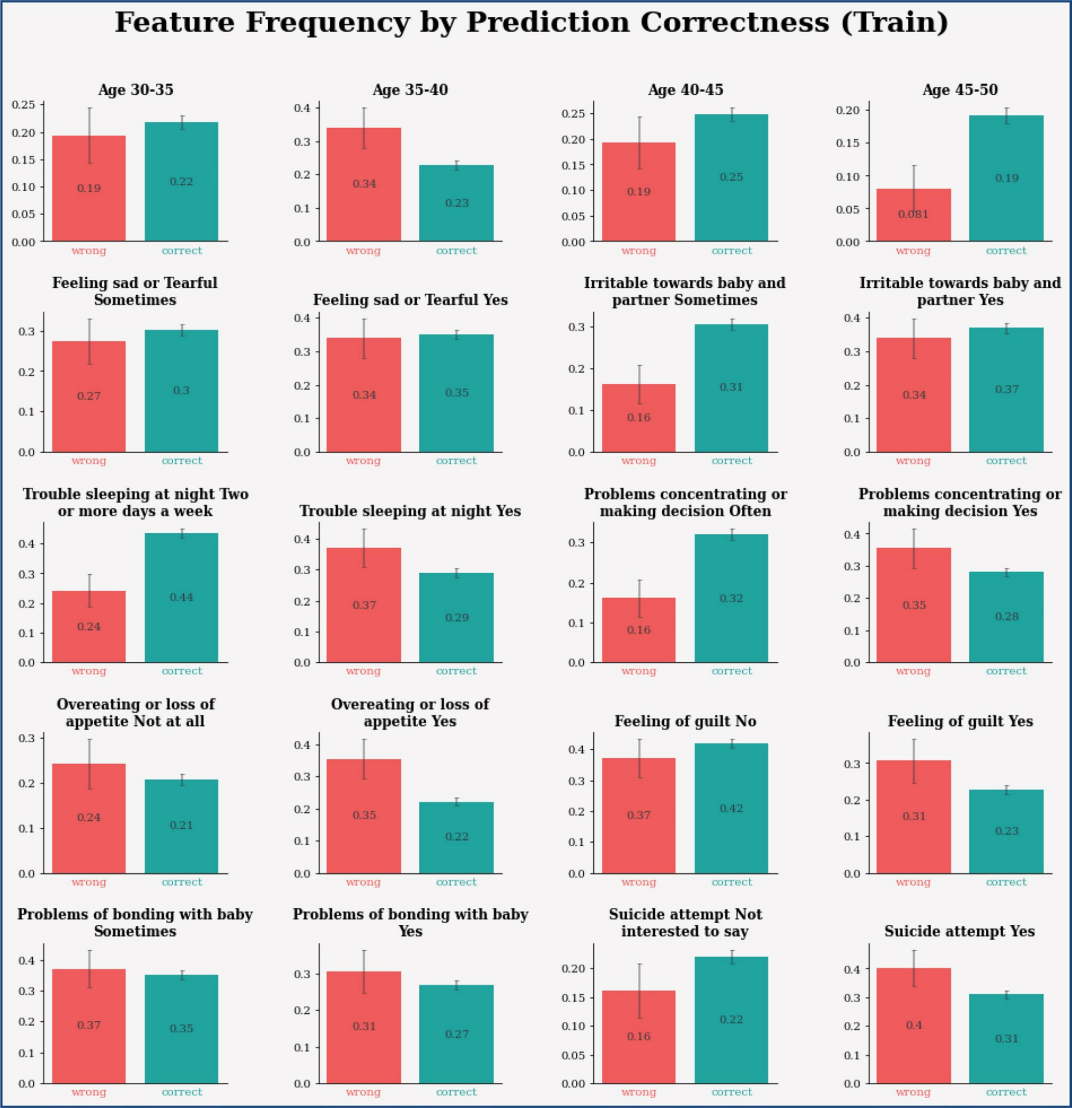
Feature frequency analysis results in training for the proposed model.

**Figure 15:**
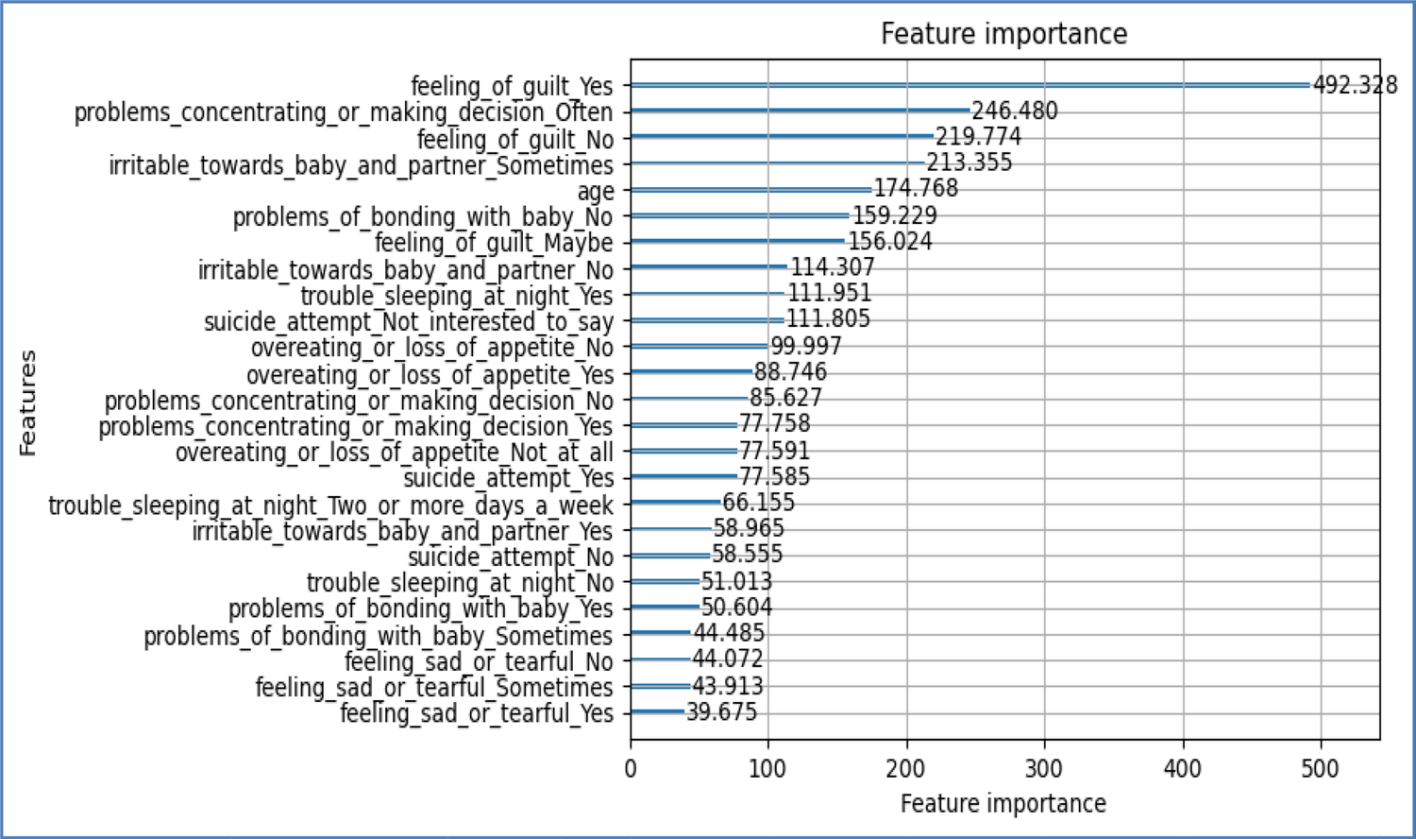
Feature importance results of the proposed model.

**Figure 16:**
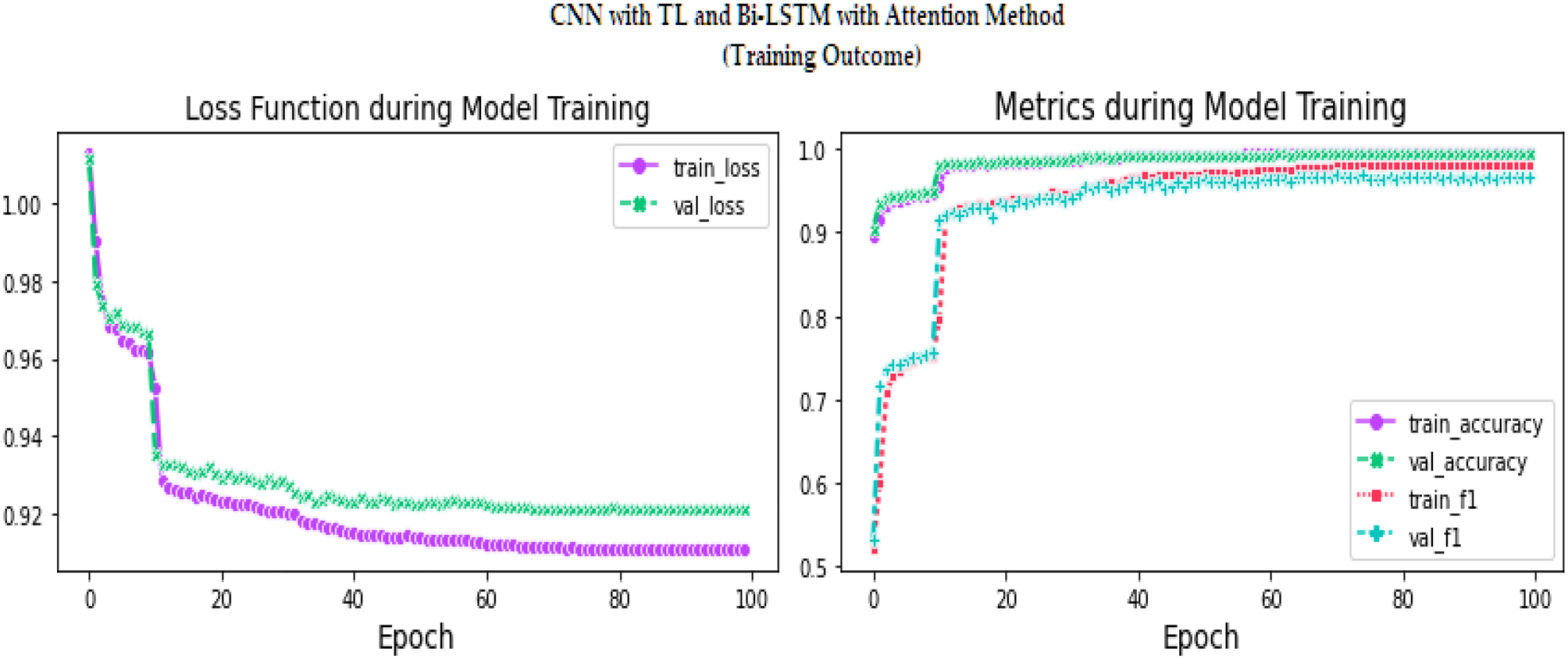
Training results for proposed model over 100 Epochs.

**Figure 17:**
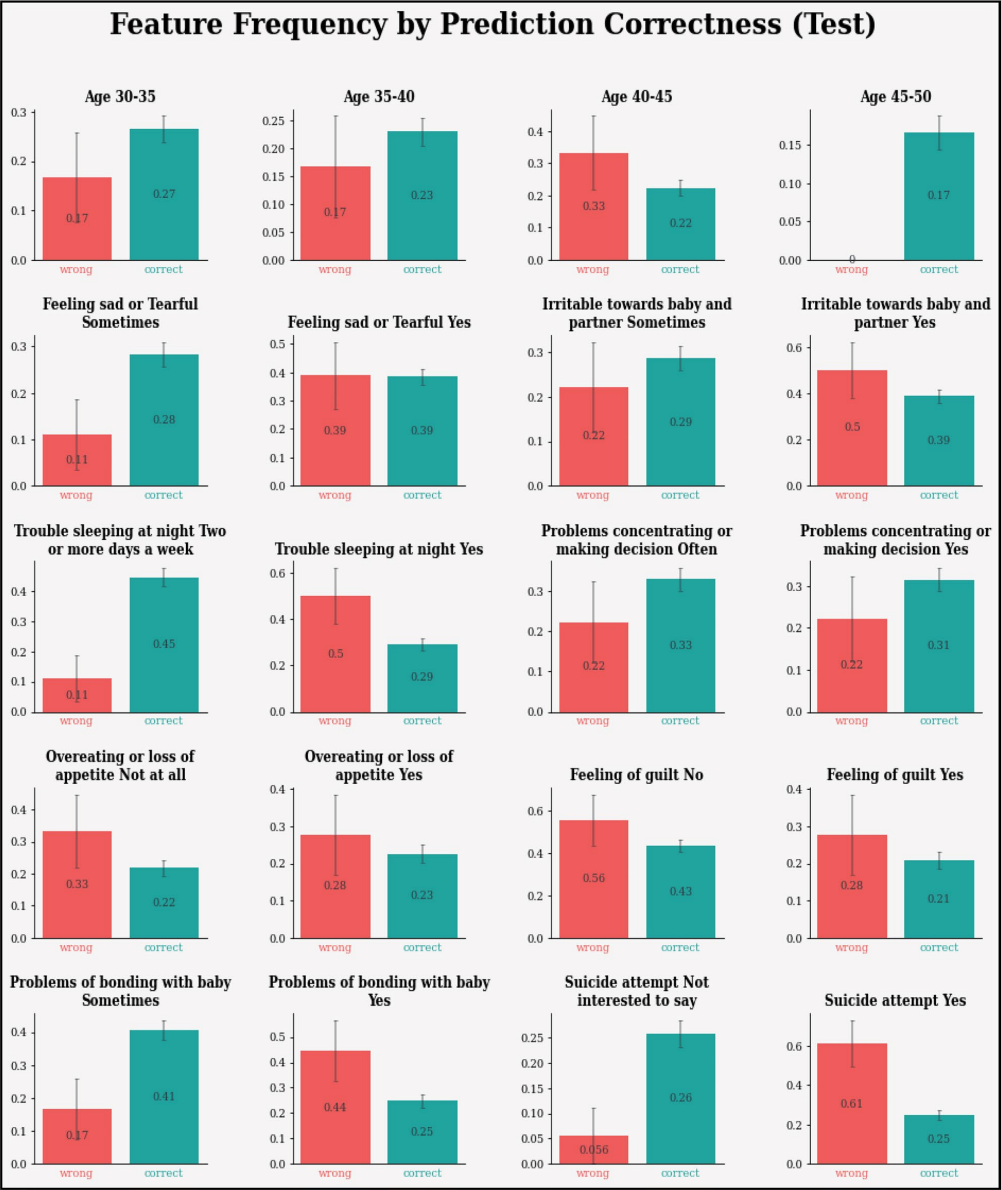
Feature frequency analysis results in testing for the proposed model.

**Figure 18:**
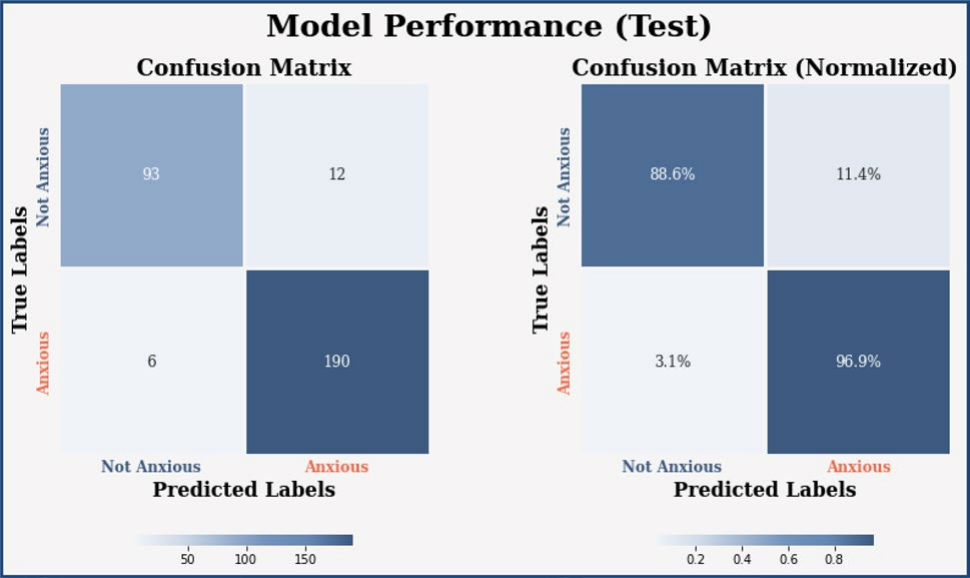
Confusion Matrix of the proposed model for the test dataset.

**Figure 19:**
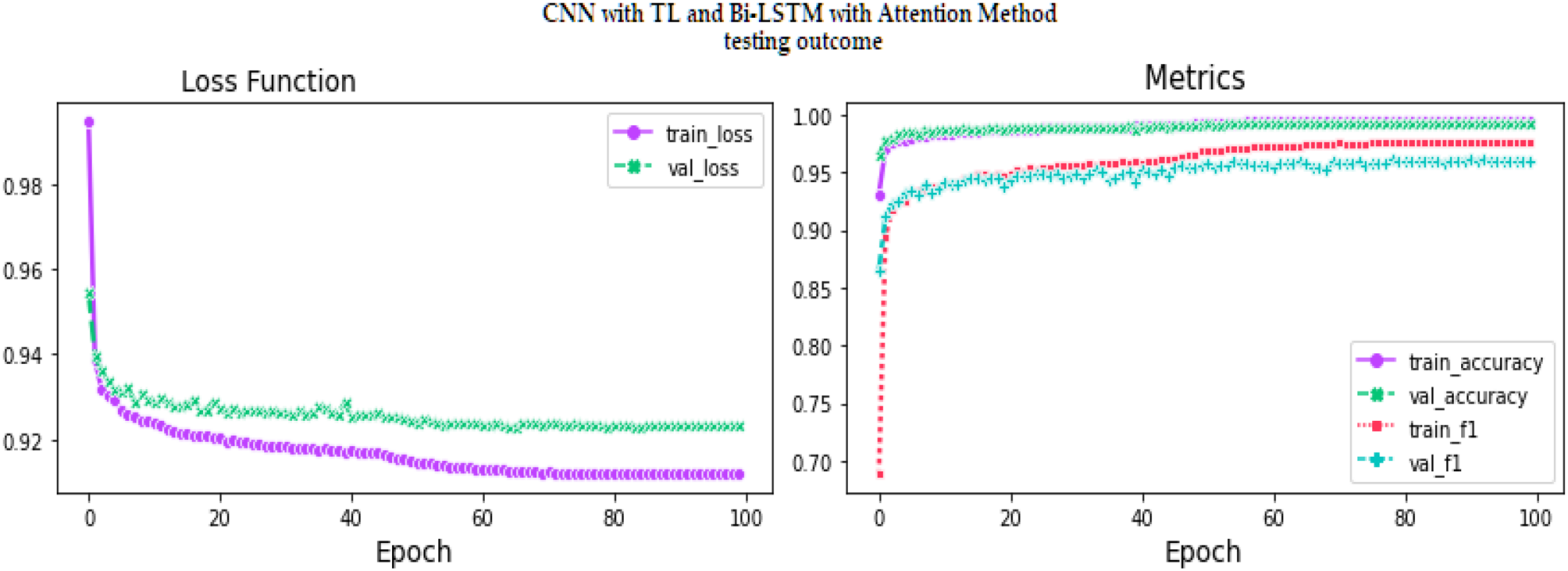
Testingresults for the Proposed model over 100 Epochs.

**Figure 20:**
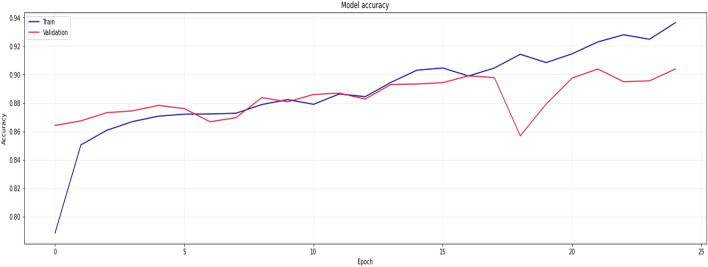
Testing results (Accuracy) for existing CNN model over 100 Epochs.

**Figure 21:**
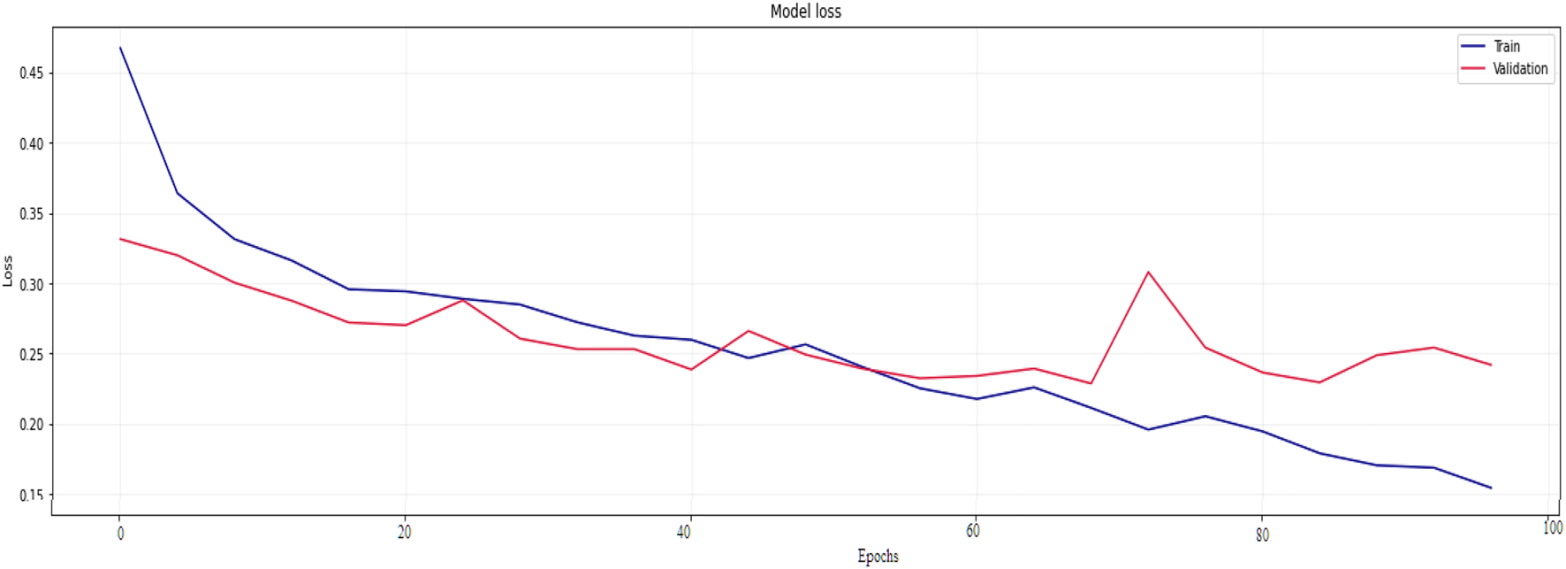
Testing results (Loss) for existing CNN model over 100 Epochs.

**Figure 22:**
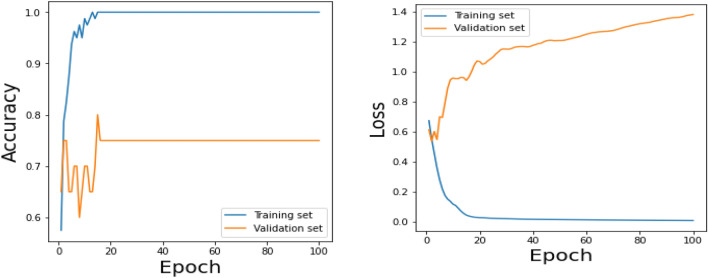
Training results (accuracy and Loss) for existing CNN-LSTM model over 100 Epochs.

**Figure 23:**
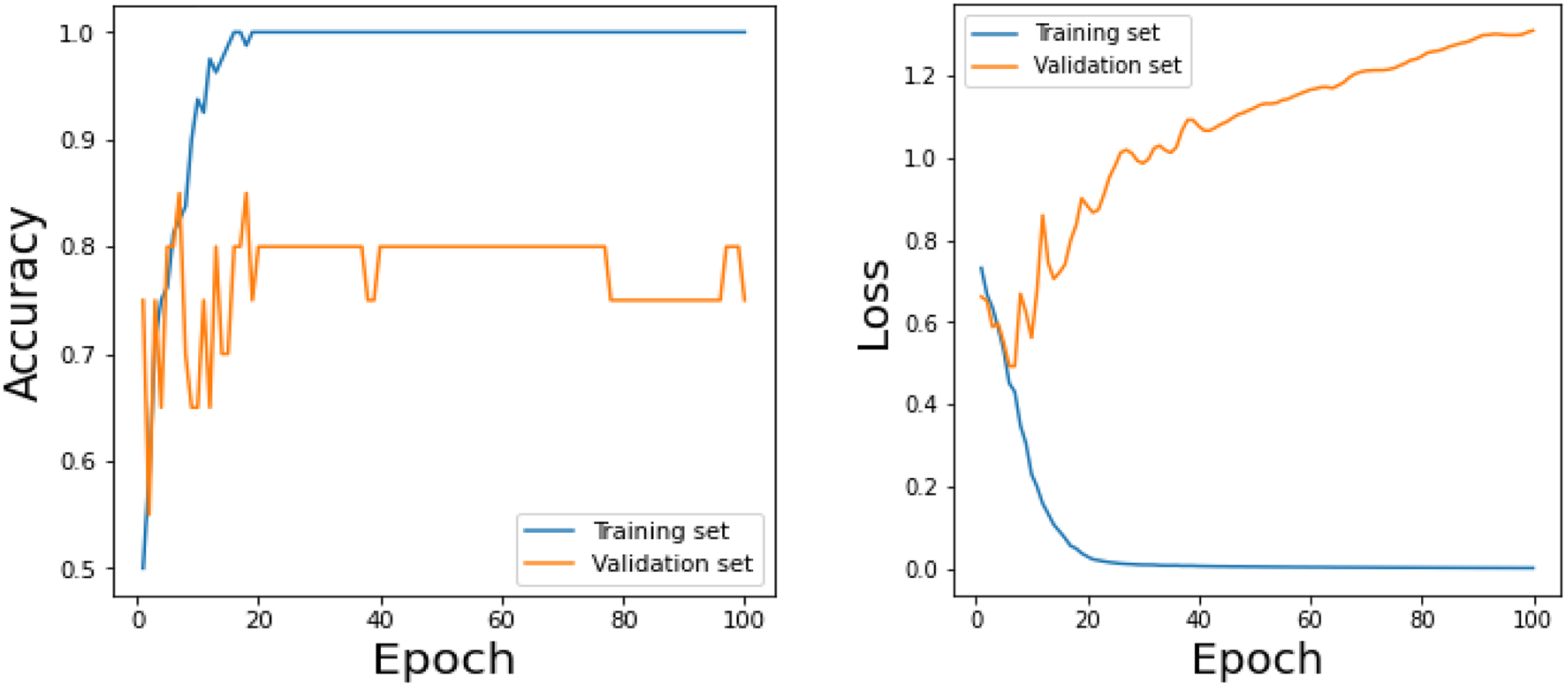
Training results (accuracy and Loss) for existing VGG-16 model over 100 Epochs.

Figures 13, 14, 15, 16, 17, 18, 19, 20, 21, 22 and 23 presents the visualization of simulation results for 100 Epochs for existing and proposed models. Figure 13 shows the Confusion Matrix Results for the proposed model on the training dataset; Fig. 14 presents the simulation results for Feature frequency analysis results in training for the proposed model. Figure 15 presents the Feature importance results of the proposed model, Fig. 16 presents the Training results for the proposed model over 100 Epochs and Fig. 17 shows the Feature frequency analysis results in testing the proposed model.

Similarly, Fig. 18 shows a Confusion Matrix of the proposed model for the test dataset. Figure 19 shows the testing results for the Proposed model over 100 Epochs, and Fig. 20 shows the Testing results (Accuracy) for the existing CNN model over 100 Epochs; Fig. 21 shows the Testing results (Loss) for the existing CNN model over 100 Epochs, Fig. 22 shows the Training results (accuracy and Loss) for existing CNN-LSTM model over 100 Epochs and Fig. 23 shows the Training results (accuracy and Loss) for existing VGG-16 model over 100 Epochs.

### Simulation 2

In simulation 2, the dataset is divided into 80%, 10%, and 10% for training, testing and validation and the results were computed for 200 epochs for proposed and existing deep learning models. Table 6 compares the results for the PPDD dataset over 200 Epochs.

**Table 6:** Results comparison for the PPDD dataset over 200 Epochs.

Technique	Accuracy %	Precision %	F1-score %	Recall %	Mean Square Error
Proposed Hybrid Model	98.188%	98.712%	98.467%	98.407%	0.01071
CNN Model	90.501%	91.741%	91.108%	90.817%	0.03191
VGG-16	92.348%	92.178%	92.478%	92.935%	0.02994
CNN-LSTM	93.741%	93.109%	93.625%	92.917%	0.01871

Table 6 presents the simulation results, comparing the proposed and existing model for the PPDD dataset over 200 Epochs. The proposed model achieved an Accuracy of 98.188%, precision of 98.172%, F1-score of 98.467%, Recall of 98.407% and Mean Square Error of 0.01071. Similarly, we have implemented existing methods on the same dataset. The existing CNN model achieved an Accuracy of 90.501%, precision of 91.741%, F1-score of 91.108%, Recall of 90.817% and Mean Square Error of 0.03191. The existing VGG-16 model achieved an Accuracy of 92.348%, precision of 92.178%, F1score of 92.478%, recall of 92.935% and Mean Square Error of 0.02994 and the existing CNN-LSTM model achieved an accuracy of 93.741%, precision of 93.109%, F1-score of 93.625%, Recall of 92.917% and Mean Square Error of 0.01871. The above experimental results prove that the proposed model performed better than existing models.

**Figure 24:**
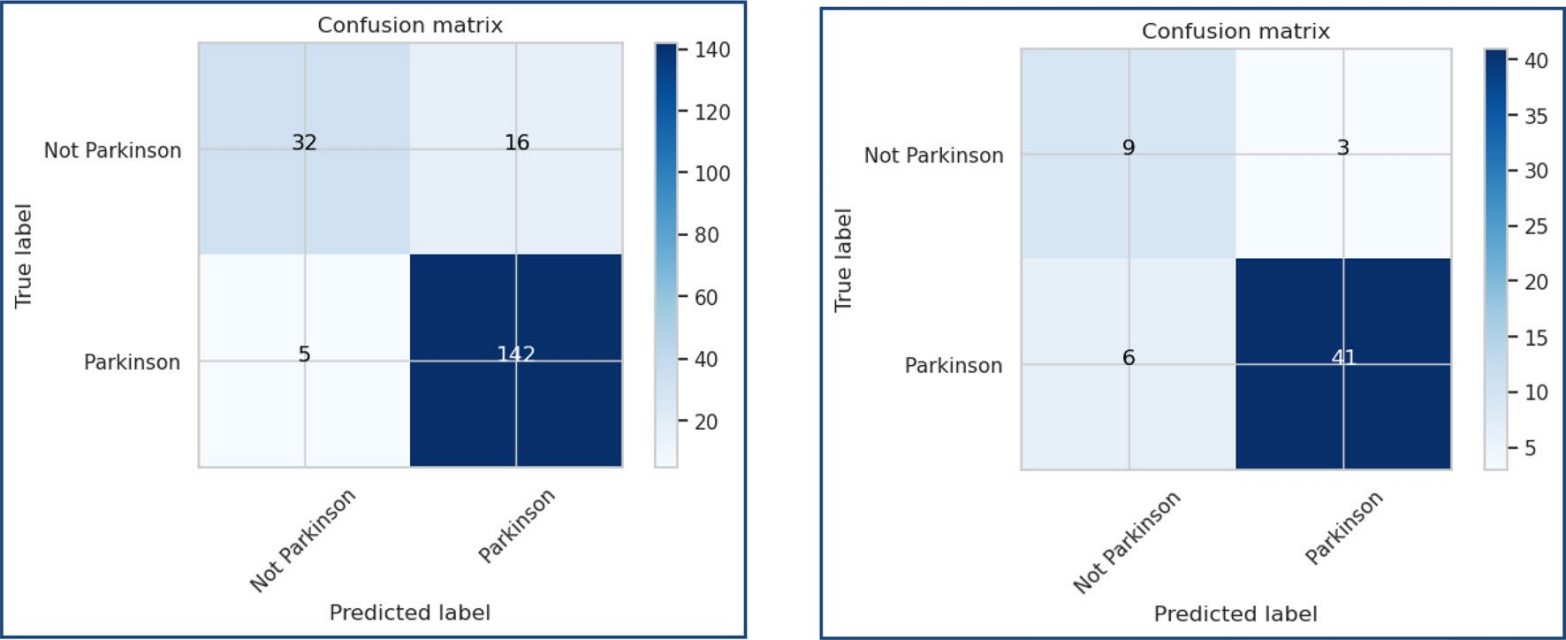
Confusion matrix for training and testing of proposed model for 200 Epochs.

**Figure 25:**
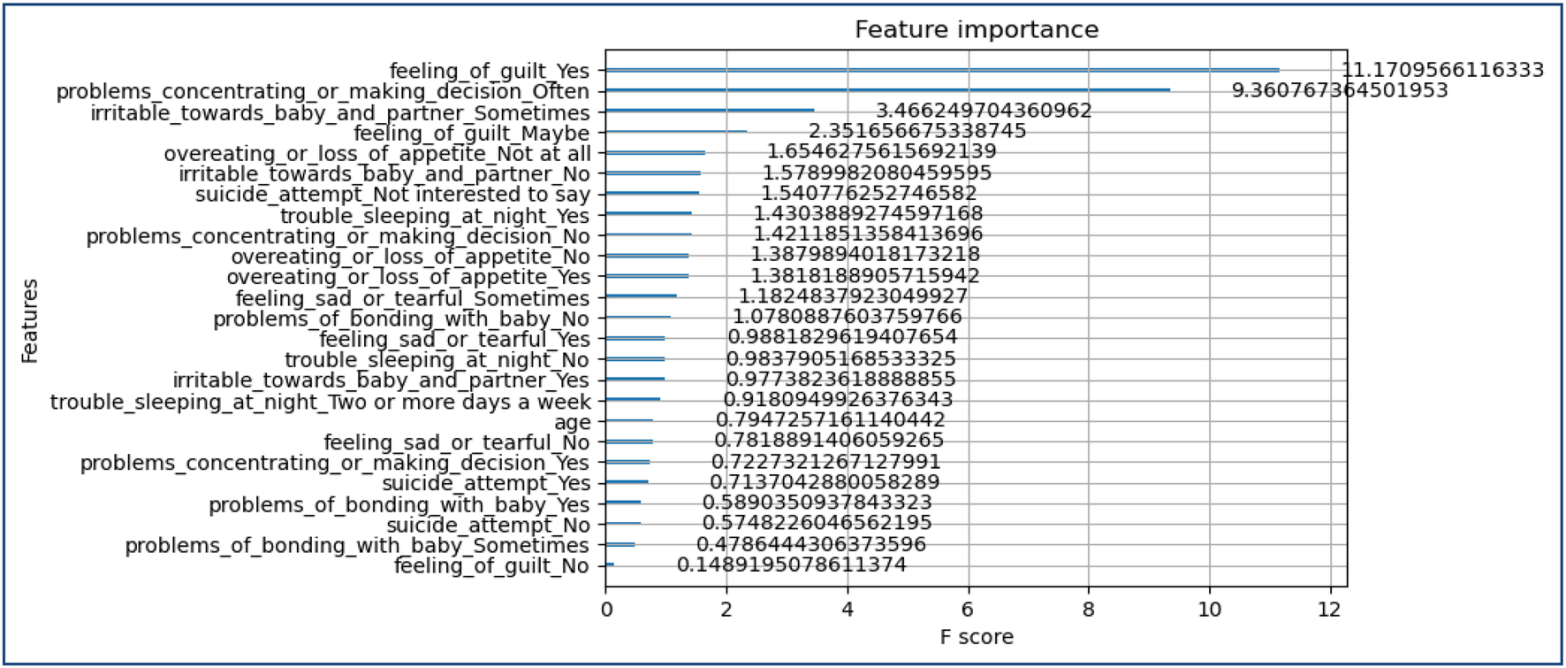
Feature importance results of the proposed model for second simulation 2.

**Figure 26:**
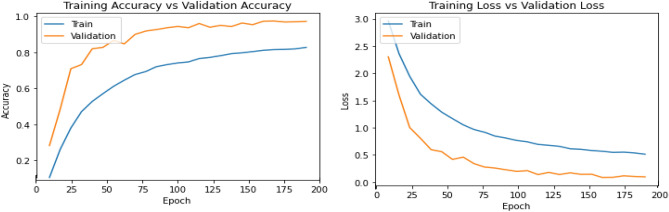
Training results (Accuracy and Loss) of the proposed model for 200 Epochs.

**Figure 27:**
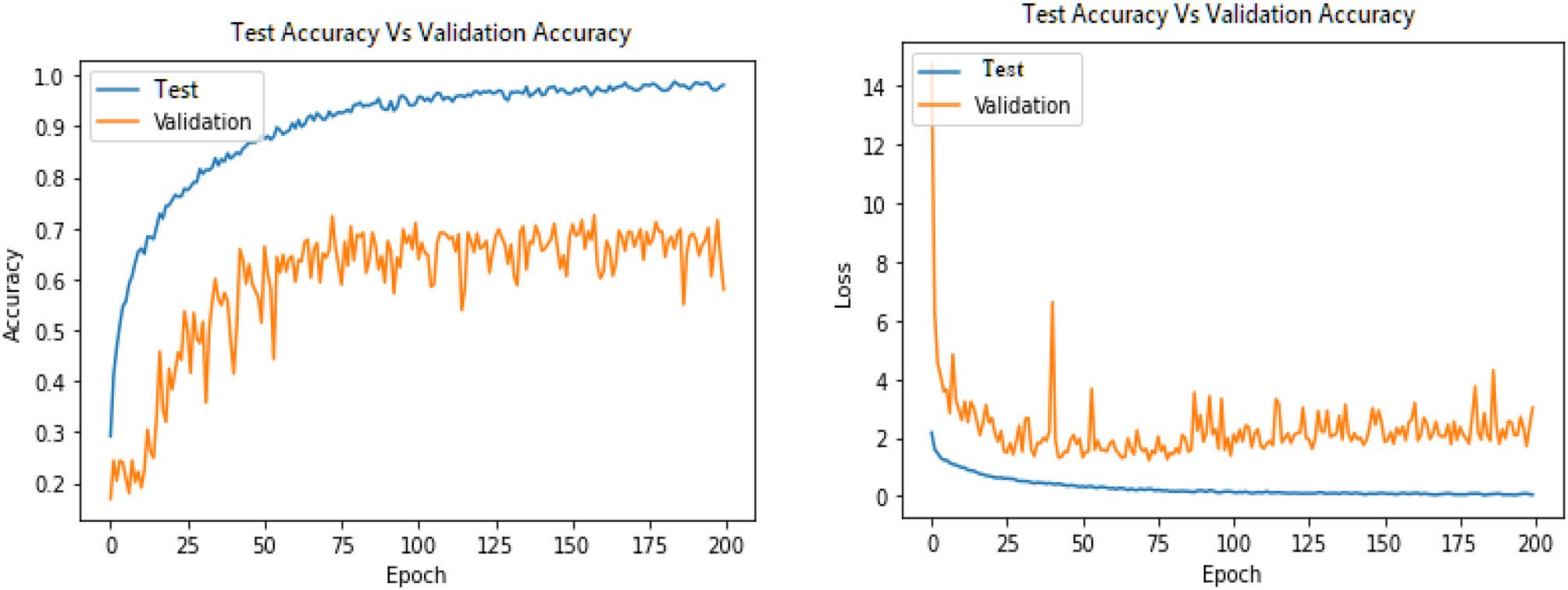
Testing results (Accuracy and Loss) of the proposed model for 200 Epochs.

Figures 24, 25, 26 and 27 show the visual representation of the proposed model. Figure 24 presents a Confusion Matrix for Training and Testing of the proposed model for 200 Epochs, and Fig. 25 shows the Feature importance results of the Proposed Model for second simulation 2. Figure 26 shows the Training results (Accuracy and Loss) of the proposed model for 200 Epochs, and Fig. 27 presents Testing results (Accuracy and Loss) for the proposed model for 200 Epochs.

### Results and discussion

The experimental results are calculated for proposed hybrid and existing models, i.e., VGG-16, CNN, and CNN-LSTM over PPDD text and audio datasets. Experimental results are calculated by applying two simulation scenarios. The simulation 1 dataset is divided into 70%, 15%, and 15% for training, testing and validation and the results were computed for 100 epochs for proposed and existing deep learning models. Table 5 presents the simulation results, comparing the proposed and Existing model for the PPDD dataset over 100 Epochs. The proposed model achieved an accuracy of 96.125%, precision of 96.152%, F1-score of 96.710%, Recall of 96.471% and Mean Square Error of 0.01121. Similarly, we have implemented the same dataset's existing methods, i.e., VGG-16, CNN and CNN-LSTM. The simulation one experimental results proved that the proposed model performed better than existing models. Figures 13, 14, 15, 16, 17, 18, 19, 20, 21, 22 and 23 presents the visualization of simulation results for 100 Epochs for existing and proposed models.

Similar To the simulation, 2 datasets are divided into 80%, 10%, and 10% for training, testing and validation, and the results were computed for 200 epochs for proposed and existing deep learning models. Table 6 presents the simulation results, comparing the proposed and Existing model for the PPDD dataset over 200 Epochs. The proposed model achieved an Accuracy of 98.188%, precision of 98.172%, F1-score of 98.467%, Recall of 98.407% and Mean Square Error of 0.01071. Similarly, we have implemented existing methods on the same PPDD dataset for 200 epochs. The simulation's two experimental results prove that the proposed model performed better than existing models. Figures 24, 25, 26 and 27 show the visual representation of the proposed mode for 200 epochs of simulation two.

- **Improved PPD Risk Factor Identification:** Implementing the proposed hybrid model enhanced the detection of risk factors associated with postpartum depression. During a thorough assessment, the model exhibited a remarkable precision of 96.152% in simulation 1 and 98.12% in simulation 2, showcasing its capacity to classify individuals at risk of PPD precisely. In addition, it attained a recall rate exceeding 96% in both scenarios, demonstrating the model’s high level of competence in identifying the most positive cases. The detection of risk factors is crucial for prompt treatment and care.
- **Improved data Feature Extraction:** The hybrid model developed in this research has demonstrated exceptional performance in feature extraction by utilizing the capabilities of CNN-based transfer learning combined with the attention strategy in the Bi-LSTM. The PPDD dataset has been effectively analyzed to identify subtle patterns. The F1-score of over 98 in simulations one and two demonstrates the model's equitable capacity to identify each PPDD positive and negative instance. Including text and audio input in addition to text has broadened the model's ability to extract features, resulting in a powerful multi-modal approach.
- **Advancement in Maternal Mental Health Research:** This research makes a substantial contribution to the continuing progress of investigation on female mental health. We have developed a novel hybrid model that combines various modalities and attention strategies. This model can be used as an effective tool for comprehending and detecting PPDD at an early stage. The accuracy value of more than 98% indicates that the model has a high accuracy level in distinguishing between positive and negative instances. This makes it a highly valuable tool in the field.
- **Enhanced Performance Measurement Parameter Outcomes:** The efficiency metrics of the proposed hybrid method are remarkable, highlighting its efficacy in PPDD detection. The proposed model attained more than 98% precision, denoting the proportion of accurately classified instances. In addition, its accuracy, recall, and F1-score exhibit its equitable capacity to minimize incorrect identifications while recording as many accurate positive cases as feasible. The elevated MSE value demonstrates the model's robust discriminatory capability, establishing it as a dependable instrument for the advanced prediction of PPDD.

The attention process in the Bi-LSTM structure is most notably successful in capturing significant characteristics in the input patterns, as demonstrated by the primarily qualitative evaluation of particular attention maps. Although the computational resources needed for training the CNN-TL system have become greater, this compromise is appropriate because of its more effective accuracy. The selection of these models is contingent upon the task’s specific demands and the resources at hand. The CNN-TL structure demonstrates superior predictive accuracy, whereas the Bi-LSTM model improves interpretability through the attention process.

## Conclusion and future works

This research introduces a new method for identifying risk factors for Postpartum Depression by utilizing deep learning and integrating multiple data types. The proposed model integrates a Convolutional Neural Network, Transfer Learning, and a Bi-LSTM-based attention mechanism. This combination provides an efficient method for identifying PPDD risk factors at an early stage. We have proven that the model accurately identifies PPDD risk factors by conducting extensive testing and validation. The experimental results are calculated for proposed hybrid and existing models, i.e., VGG-16, CNN, and CNN-LSTM over PPDD text and audio datasets. Experimental results are calculated by applying two simulation scenarios. The Simulation 1 dataset is divided into 70%, 15%, and 15% for training, testing and validation. Similar To the simulation, 2 datasets are divided into 80%, 10%, and 10% for training, testing and validation, and the results were computed for 200 epochs; the results were computed for 100 and 200 epochs for proposed and existing deep learning models. The proposed model achieved an Accuracy of 96.125%, precision of 96.152%, F1-score of 96.710%, recall of 96.471% and Mean Square Error of 0.01121 for 100 epochs in simulation one and Accuracy of 98.188%, precision of 98.172%, F1-score of 98.467%, Recall of 98.407% and Mean Square Error of 0.01071 for 200 epochs in simulation two for proposed model. The simulation’s two experimental results prove that the proposed model performed better than existing models.

Furthermore, our findings have demonstrated exceptional accuracy, recall, precision and F1 score metrics, highlighting the model’s proficiency in accurately detecting the two positive and negative instances. The proposed method’s multi-modal design, incorporating textual and audio data, deepens our comprehension of PPDD risk variables. This not only enhances the ability to detect problems at an early stage but also provides opportunities for implementing more comprehensive assistance and intervention methods for mothers who are at risk. The outstanding performance of our model, combined with its multi-modal approach, shows great potential for enhancing maternal mental health. Subsequent efforts mainly improve and broaden our model, augment its comprehensibility, and seamlessly incorporate it into healthcare protocols, ultimately enhancing mothers worldwide.

## The findings of our study have significant implications and present numerous stimulating avenues for future research.

- Increased and Varied Datasets: By augmenting our dataset with a greater quantity and wider variety of clinical data and patient interviews, we can enhance the model's performance and ability to apply its knowledge to new situations.
- Interpretability: It is crucial to develop techniques that allow us to understand and explain how models work. We aim to investigate techniques for representing attention mechanisms and feature importance to enhance predictions' interpretability.
- Real-time Monitoring: Modifying the model to enable real-time monitoring can offer prompt assistance and intervention. Incorporating tele-health platforms and mobile applications is a potential plan for the future.
- Multi-modal Data Fusion**: Further exploration of integrating diverse data types, such as visual and physiological data; will enhance our comprehension of risk factors associated with PPDD.
- Longitudinal Analysis: Conducting longitudinal studies to track risk factors for PPDD over time will offer valuable insights into the changing nature of these factors and the efficacy of interventions.
- Privacy and Ethical Considerations: We emphasize the significance of implementing strong data anonymization techniques and ethical considerations. Subsequent efforts will prioritize enhancing measures to protect privacy.
- Clinical Validation: It is crucial to collaborate with healthcare professionals to validate the model and integrate it into clinical practice to ensure its practical effectiveness in real-world settings.

## Data Availability

This research utilizes an online PPDD dataset. The dataset will also be available upon individual request to the corresponding author.
